# Structural Analysis of Virus Regulatory N6-Methyladenosine (m6A) Machinery of the Black Flying Fox (*Pteropus alecto*) and the Egyptian Fruit Bat (*Rousettus aegyptiacus*) Shows Evolutionary Conservation Amongst Mammals

**DOI:** 10.3390/genes15111361

**Published:** 2024-10-23

**Authors:** Asmaa Nasr, Nikki Copeland, Muhammad Munir

**Affiliations:** 1 Division of Biomedical and Life Sciences, Lancaster University, Lancaster LA1 4YG, UK or a.nasr@lancaster.ac.uk (A.N.); n.cpeland@lancaster.ac.uk (N.C.); 2 Department of Zoonoses, Faculty of Veterinary Medicine, Cairo University, Giza 12211, Egypt

**Keywords:** N6-methyladenosine (m6A), bats, *Pteropus alecto*, *Rousettus aegyptiacus*, *Homo sapiens*

## Abstract

Background: N6-methyladenosine (m6A) is an abundant RNA epitranscriptomic modification in eukaryotes. The m6A machinery includes cellular writer, eraser and reader proteins that regulate m6A. *Pteropus alecto* (*P. alecto*) (the Australian black flying fox) and *Rousettus aegyptiacus* (*R. aegyptiacus*) (the Egyptian fruit bat) are bats associated with several viral zoonoses yet neglected in the field of m6A epigenetics studies. Objectives: This study utilises various bioinformatics and in silico tools to genetically identify, characterise and annotate the m6A machinery in *P. alecto* and *R. aegyptiacus*. Methods: A range of bioinformatic tools were deployed to comprehensively characterise all known m6A-associated proteins of *P. alecto* and *R. aegyptiacus.* Results: Phylogenetically, the m6A fat mass and obesity-associated protein (FTO) eraser placed the order Chiroptera (an order including all bat species) in a separate clade. Additionally, it showed the lowest identity matrices in *P. alecto* and *R. aegyptiacus* when compared to other mammals (74.1% and 72.8%) and *Homo sapiens* (84.0% and 76.1%), respectively. When compared to humans, genetic loci-based analysis of *P. alecto* and *R. aegyptiacus* showed syntenic conservation in multiple flanking genes of 8 out the 10 m6A-associated genes. Furthermore, amino acid alignment and protein tertiary structure of the two bats’ m6A machinery demonstrated conservation in the writers but not in erasers and readers, compared to humans. Conclusions: These studies provide foundational annotation and genetic characterisation of m6A machinery in two important species of bats which can be exploited to study bat–virus interactions at the interface of epitranscriptomics.

## 1. Introduction

Several epigenetic modifications have been discovered in both DNA and RNA, although modifications in the DNA are limited compared to the large number present in the RNAs (~170) [1]. N6-methyladenosine (m6A) is an RNA post-transcriptional modification that involves the addition of a methyl group to the sixth nitrogen atom of the adenosine (m6A) nucleotide base [2,3]. It is evolutionarily conserved and participates in several processes associated with cell physiology and pathology. Additionally, the role of m6A was verified in the replication and immune defence against several DNA and RNA viruses [4,5,6,7,8,9]. Messenger RNA (mRNA) is the type of RNA most frequently subjected to this type of modification [10], although other RNA types can be methylated as well, including transfer RNAs (tRNAs) [11], ribosomal RNAs (rRNAs) [12], microRNAs (miRNAs) [13] and long non-coding RNAs (lncRNAs) [14]. Indeed, m6A is common within the transcriptome, accounting for approximately 50% of all methylated ribonucleotides [15].

m6A is a reversible modification, coordinated by three well-identified m6A regulators: writers, erasers and readers. Writers are located in the nucleus, and they are responsible for the installation of the m6A modification around the long internal exons, upstream sites of the stop codons, and 3′ and 5′ untranslated terminal regions (UTRs) of the transcripts [15,16,17,18]. Methyltransferase like-3 (METTL3) is the main catalytic enzyme that adds the methyl group to adenosine with the aid of methyltransferase like 14 (METTL14), which forms a heterodimer with METTL3 [19]. Wilms′ Tumor 1-associating Protein (WTAP), is another writer auxiliary protein responsible for recruiting the METTL3–METTL14 complex to the site of the m6A methylation, the nuclear speckles [20]. m6A erasers are demethylases that remove the methyl group from the modified transcripts. So far, two erasers with comparable activity have been identified including fat mass and obesity-associated protein (FTO) [21] and α-ketoglutarate-dependent dioxygenase alkB homolog 5 (ALKBH5) [22]. Both are AlkB family proteins that are distinguished by the presence of the AlkB domain or jelly roll motif responsible for catalysing the oxidation of m6A into adenosine [23]. Readers are a network of cytoplasmic and nuclear proteins that can bind to the m6A-containing transcripts upon their nuclear export, regulating their fates. Several proteins carrying the YT521-B homology (YTH) domain have been identified and thus constitute the largest group of readers. This class comprises five major proteins directing the output of the methylated mRNA, including YTHDF1, YTHDF2, YTHDF3, YTHDC1 and YTHDC2 [24,25].

Writers, erasers and readers of m6A have been extensively studied in mammalian species; however, they have not been genetically described in any bat species. These flying mammals of the order Chiroptera make up about one-fifth of all mammalian species on earth, representing an important portion of the ecosystem, and are known for their unique immune system [26]. In general, bats are classified into two suborders, Microchiroptera (microbats) and Megachiroptera (microbats). *P. alecto* (the Australian black flying fox) and *R. aegyptiacus* (the Egyptian fruit bat) are two megabats classified within the Pteropodidae family [27,28]. *P. alecto* has been widely investigated in epidemiological research due to its natural reservoir potential for Hendra viruses [26,29,30]. Additionally, *P. alecto* plays an important role in the transmission of many other zoonotic viruses, including Australian bat lyssavirus and Japanese encephalitis viruses [31,32]. *R. aegyptiacus* is the natural reservoir of Marburg filovirus [33]. Additionally, during the COVID-19 pandemic, *R. aegyptiacus* was shown to be susceptible to SARS-CoV-2 infection [34]. Despite the availability of genomic annotation in these two bat species [35,36], they remain neglected in the field of epitranscriptomics. This study uses in silico structural and bioinformatic approaches to annotate the m6A machinery (m6A readers, writers and erasers) of *P. alecto* and *R. aegyptiacus* in comparison to *H. sapiens*.

## 2. Materials and Methods

### 2.1. Databases and Sequence Collection

Protein sequences of the m6A machinery of *P. alecto*, *R. aegyptiacus* and *H. sapiens* were accessed and downloaded in FASTA format from the National Center for Biotechnology Information (NCBI) protein database https://www.ncbi.nlm.nih.gov/protein/ (accessed on 22 July 2022). To understand the evolution and similarity difference of the m6A machinery of *P. alecto* and *R. aegyptiacus* in comparison to different animals, sequences for various orthologue classes including Mammalia, Aves, Reptilia, Amphibia and Osteichthyes were collected. Accession numbers for the *H. sapiens*, *R. aegyptiacus* and *P. alecto* sequences used in this study are shown in Table 1.

### 2.2. Sequence Alignment and Phylogenetic Analysis

Prior to the phylogenetic analysis and identity matrix calculation, amino acid sequences of the retrieved orthologues were trimmed to improve the quality of the alignment and aligned using the MUSCLE algorithm of Geneious Prime 2022.1 software http://www.geneious.com/ (accessed on 22 July 2022). The phylogeny of the aligned sequences was determined using the maximum-likelihood method of PhyML.30 software provided by the ATGC server http://www.atgc-montpellier.fr/phyml/ (accessed on 22 July 2022) [37]. A total of 1000 bootstrap supports were inferred to ensure the robustness of the trees. On the generation of the trees, all sequences were visualised, marked and grouped according to their classes using MEGA 11 Version 11.0.11 software [38].

### 2.3. Pairwise Identity Calculation and SDT Graphical Presentation

Sequences used in the phylogenetic tree analysis were also assessed for pairwise identity matrix by applying the MUSCLE alignment tool of Sequence Demarcation Tool Version 1.2 (SDTv1.2) software [39]. The generated graphs were then annotated to display the clustering of identical and differing groups.

### 2.4. Genomic Comparisons and Genetic Synteny Analyses

For genomic comparisons, genomes and annotations of *R. aegyptiacus*, *P. alecto* and *H. sapiens* were downloaded from the NCBI in FASTA and GFF formats (https://www.ncbi.nlm.nih.gov/genome/, accessed on 22 July 2022) (Table 2). The downloaded files were used as inputs for the MCScanX algorithm of TBtools software Version 1.106 [40], and the m6A chromosomes and scaffolds were determined on the generation of the graphs using the same software. For evaluating the genetic synteny, genes of m6A-related proteins along with 6 flanking genes were determined in three species using the NCBI Genome Data Viewer (GDV) https://www.ncbi.nlm.nih.gov/genome/gdv (accessed on 22 July 2022). Accordingly, figures of the genes along with their orientations and annotated chromosomes and scaffolds were generated.

### 2.5. Amino Acid Alignment and Tertiary Protein Structure Comparison

For a comparison of the amino acids of m6A machinery between humans and the two bat species, Clustal W alignment was performed using BioEdit 7.2 software on the FASTA sequences provided in Table 1. For comparing the 3D structures, m6A proteins of *H. sapiens* were retrieved from the Protein Data Bank (PDB) https://www.rcsb.org/ (accessed on 22 July 2022) (Table 3), while the proteins of *R. aegyptiacus* and *P. alecto* were submitted for prediction using the SWISS-MODEL homology modelling server https://swissmodel.expasy.org/ (accessed on 22 July 2022). To increase the accuracy of the SWISS-MODEL template and model selections, the m6A machinery of the two bats was submitted in the form of domains and again as whole protein sequences using the SWISS-MODEL sequence-based mode. For the construction of YTHDC1 ligand–amino acid interaction predictions, the two bat YTHDC1 proteins were input to the server along with the human PDB corresponding protein using the user template project mode. Identity, coverage and Global Model Quality Estimation (GMQE) were used to select the best templates, while GMQE and QMEANDisCo were the most important parameters for choosing the best models. All models were visualised and edited by PyMOL 2.5 https://pymol.org/2/ (accessed on 22 July 2022).

For establishing the YTH domain sequence alignment with secondary structure information, the ESPript 3.0 server https://espript.ibcp.fr/ESPript/ESPript/ (accessed on 22 July 2022) was used [41]. In this case, the alignment of the YTH domain sequences, which was performed by ClustalW of BioEdit 7.2, and the secondary structure YTHDF2 PDB entry code 4WQN was submitted to the server. Protein domains, important structures and critical amino acids were determined and annotated in the figures accordingly.

## 3. Results

### 3.1. Evolutionary Analysis of R. aegyptiacus and P. alecto m6A-Associated Proteins Revealed Mammalian Clustering

**Figure 1:**
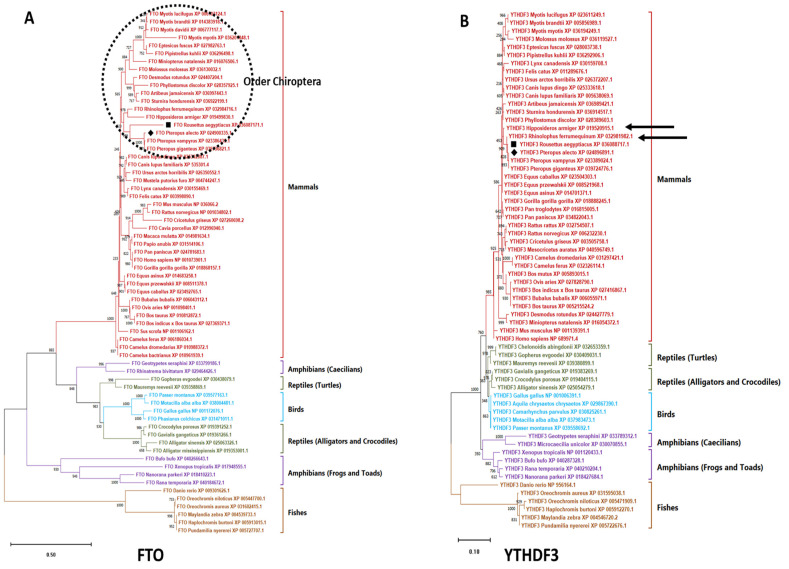
Phylogenetic trees of FTO and YTHDF3 orthologues. (**A**) The phylogram of the FTO orthologues. The position of order Chiroptera in the tree is marked by a black dashed circle. (**B**) The phylogram of the YTHDF3 orthologues. The members representing family Rhinolophidae and Hipposideridae are marked by black arrows. m6A proteins of *P. alecto* and *R. aegyptiacus* are marked with solid diamond and cube shapes in the trees, respectively.

In order to characterise the writers, erasers and readers of *R. aegyptiacus* and *P. alecto*, we compared their evolution against those of different additional mammalian (bats, carnivores, equines, ungulates, rodents, primates) and non-mammalian (birds, reptiles, amphibian, fishes) species. Overall, based on the phylogenetic analysis, the m6A machinery of *R. aegyptiacus* and *P. alecto* were positioned in the mammalian cluster, where they were grouped as one separate clade encompassing other members of the Pteropodidae family (*P. vampyrus* and *P. giganteus*). The Pteropodidae clade in the YTHDF3 tree and Rhinolophidae (*Rhinolophus ferrumequinum*) (Rhinolophidae) shared a common ancestor, while they, as well as Hipposideridae (*Hipposideros armiger*), were descendants from another further ancestor. Furthermore, the FTOs of the order Chiroptera (the order of all bat species) were represented in the tree as a separate distinct clade yet within the superorder Laurasiatheria (the superorder of equines, carnivores and ungulates) (Figure 1A,B and Figures S1–S4).

### 3.2. R. aegyptiacus and P. alecto m6A Machinery Displayed High Amino Acid Identity to Mammals and H. sapiens

**Figure 2:**
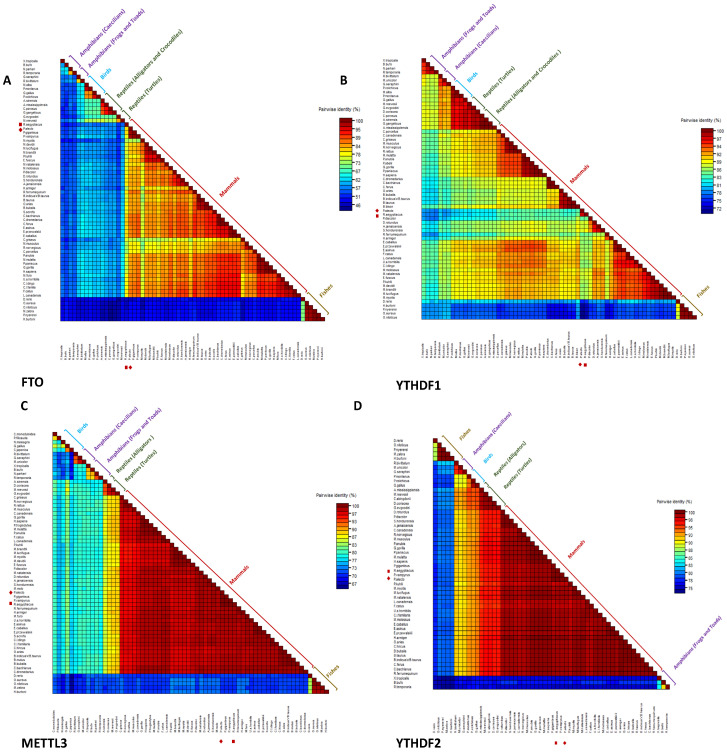
Identity matrices of FTO, YTHDF1, METTL3 and YTHDF2 orthologues. (**A**) The identity matrix scores of the FTO orthologues. (**B**) The identity matrix scores of the YTHDF1 orthologues. (**C**) The identity matrix scores of the METTL3 orthologues. (**D**) The identity matrix scores of the YTHDF2 orthologues. m6A proteins of *P. alecto* and *R. aegyptiacus* are marked with solid diamond and cube shapes, respectively.

Next, we compared the amino acid identities of the two Pteropodidae and the aforementioned mammalians and non-mammalian species, with a focus on the mammalian and human identities. In terms of the mammalian proteins’ identity, the amino acid matrix scores of the demethylase FTO among the 10 m6A regulators recorded the lowest similarity in both *P. alecto* and *R. aegyptiacus* (74.1% and 72.8%, respectively) (Figure 2A), followed by the YTHDF1 (84.2% and 83.1%, respectively) (Figure 2B), whereas the highest similarity in both species was obtained in METTL3 and YTHDF2 in the two comparisons (100%) (Figures 2C and 2D, respectively). In contrast, when *P. alecto* and *R. aegyptiacus* were compared to *H. sapiens*, YTHDF2 exhibited the highest similarity in both bats (99.3%) (Figure 2D), while FTO demonstrated the opposite for the *P. alecto* and *R. aegyptiacus* (84.0% and 76.1%, respectively) (Figure 2A).

### 3.3. Comparative Genomics of P. alecto and R. aegyptiacus Showed Complexity While the Genetic Synteny Revealed Conservation

**Figure 3:**
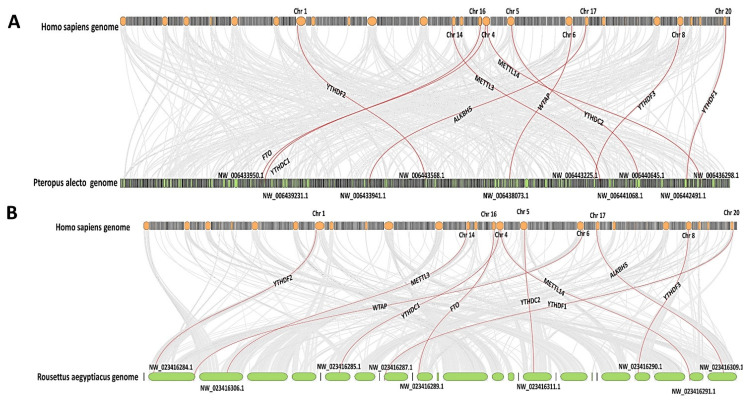
Comparative genomics of *H. sapiens*, *P. alecto*, and *R. aegyptiacus*, (**A**) Genome alignment of *H. sapiens* and *P. alecto*. (**B**) Genome alignment of *H. sapiens* and *R. aegyptiacus*. Genes of m6A are highlighted in red.

To investigate the genetic relatedness of the m6A regulators of *P. alecto* and *R. aegyptiacus* to those of *H. sapiens*, we aligned the whole genome of the two bats against that of humans with a focus on the areas of the m6A machinery and their neighbouring genes. Genes for the m6A proteins in humans were situated on chromosomes 1, 4, 5, 6, 8, 14, 16, 17 and 20, and the fourth chromosome was noted to carry METT14 and YTHDC1. On the other hand, genetic elements of the *P. alecto* and *R. aegyptiacus* m6A regulators were located on various unplaced scaffolds (Figures 3A and 3B, respectively).

**Figure 4:**
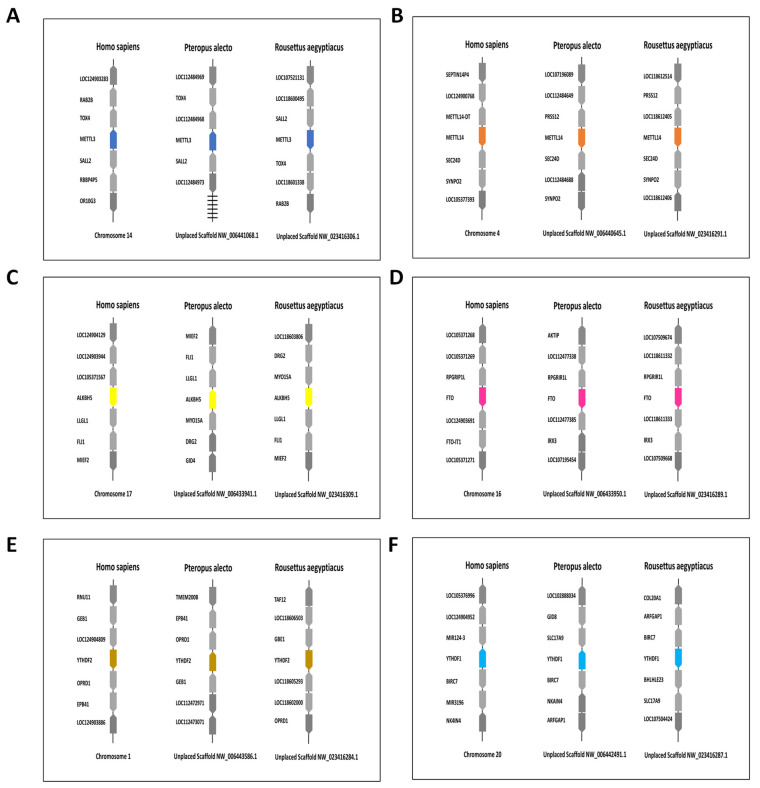
Genetic syntenic comparisons of METTL13, METTL14, FTO, ALKBH5, YTHDF1 and YTHDF2 of *H. sapiens*, *P. alecto* and *R. aegyptiacus*. (**A**) Genetic synteny of METTL13 among *H. sapiens*, *P. alecto* and *R. aegyptiacus*. (**B**) Genetic synteny of METTL14 among *H. sapiens*, *P. alecto* and *R. aegyptiacus*. (**C**) Genetic synteny of FTO among *H. sapiens*, *P. alecto* and *R. aegyptiacus*. (**D**) Genetic synteny of ALKBH5 among *H. sapiens*, *P. alecto* and *R. aegyptiacus*. (**E**) Genetic synteny of YTHDF1 among *H. sapiens*, *P. alecto* and *R. aegyptiacus*. (**F**) Genetic synteny of the YTHDF2 among *H. sapiens*, *P. alecto* and *R. aegyptiacus*.

Loci of the bats’ writer complex were noted to carry several coding genes similar to the human genome. The METTL13 loci carried *TOX* high mobility group box family member 4 (*TOX4*) and spalt-like transcription factor 2 (*SALL2*) genes (Figure 4A), and the expression of those genes was changed in the human acute lymphocytic leukaemia [42]. The METTL14 loci carried synaptopodin 2 (*SYNPO2*), where a mutation looped between the first intron of this gene and the METTL14 promotor was linked to increased susceptibility to ovarian cancers in humans (Figure 4B) [43]. Superoxide dismutase 2, mitochondrial *(SOD2*) was observed in the vicinity of the WTAP loci (Figure S6A). A single mutation in WTAP was found to increase the expression of *SOD2* which was linked to the healthy status of other tissues adjacent to bladder cancer in humans [44].

For analysis of the m6A eraser loci, the human fat metabolism regulatory gene, protein phosphatase 1, and regulatory subunit 134 (*RPGRIR1L*) were found to be conserved between human and bat FTO loci. FTO upregulates *RPGRIR1L* in humans, increasing the lean body mass; furthermore, Iroquois Homeobox 3 (*IRX3)* which was also upregulated by FTO in human obesity [45], was found on the same loci in bats (Figure 4C). Nevertheless, the human *IRX3* was found to be located further from FTO than it is in bats. Similarly, *LLGL* Scribble Cell Polarity Complex Component 1 (*LLGL1*) and Mitochondrial Elongation Factor 2 (*MIEF2*) were present on the bats’ ALKBH5 loci (Figure 4D). The expression of the *LLGL1* gene was found to be upregulated by ALKBH5 in xenograft tumours [46], while ALKBH5 and *MIEF2* were upregulated together in the T regulatory cells of metastatic melanoma [47].

In the comparison of the genetic synteny of the readers’ loci, the YTHDF loci showed several genes that were maintained across humans and the two Chiroptera species. Such examples of conservation could be seen in Baculoviral IAP Repeat Containing 7 (*BIRC7*) and Sodium/Potassium Transporting ATPase Interacting 4 (*NK4IN4*) of the YTHDF1 loci (Figure 4E) and Glucocorticoid Modulatory Element Binding Protein 1 (*GMEB1*) of the YTHDF2 loci (Figure 4F). Indeed, mutations in the YTHDF2 were linked to the lower expression of *BIRC7* and *NK4IN4* in human hepatoblastoma [48], and genetic duplication of *GMBE1* and YTHDF2 was noted in cases of human megalencephaly, suggesting them as candidate genes for this disease [49]. The YTHDF3 loci of humans and bats were noted to carry α Tocopherol Transfer Protein (*TTPA*) (Figure S6B); however, we could not find a publication that links the YTHDF3 to *TTPA* in humans. Neither bats’ YTHDC1 nor bats’ YTHDC2 loci had the same genes found on the human counter ones (Figure S6C,D).

### 3.4. P. alecto and R. aegyptiacus m6A Proteins Revealed Structural Conservation Among Writers, Not Erasers or Readers

In a trial performed to anticipate the structure and function of *P. alecto* and *R. aegyptiacus* m6A proteins during m6A processing, we adopted amino acid sequence alignment and 3D protein structures and compared them to those of *H. sapiens*.

**Figure 5:**
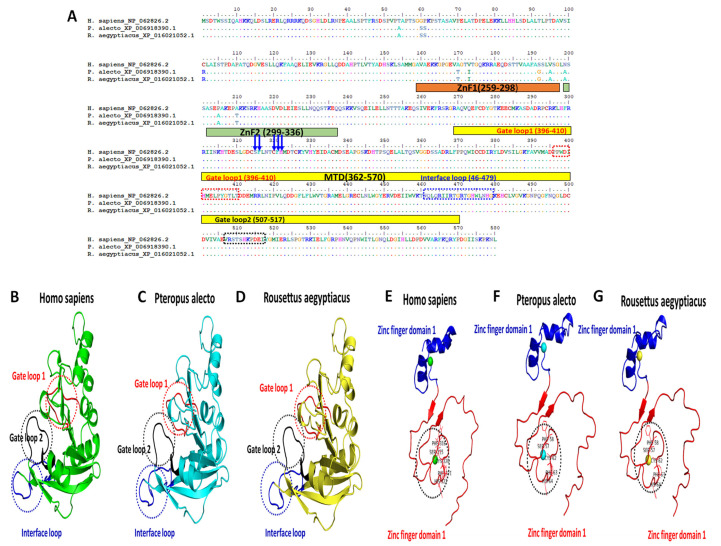
Alignment and protein structure comparisons of *H. sapiens*, *P. alecto* and *R. aegyptiacus* METTL3. (**A**) Amino acid sequence alignment of METTL3 proteins of *H. sapiens*, *P. alecto* and *R. aegyptiacus*. The ZnF1 and ZnF2 are highlighted in solid orange and green rectangles, respectively. The conserved ZnF2 hydrophobic residues (Ser315, Phe316, Cys320, Phe321 and His322) are marked with blue arrows. The METTL3 MTD is labelled with a solid yellow rectangle. Amino acids of gate loop 1, gate loop 2 and interface loop are highlighted in red, black and blue dashed frames, respectively. Overall structural comparison of METTL3 MTD among (**B**) *H. sapiens*, (**C**) *P. alecto* and (**D**) *R. aegyptiacus*. Gate loop 1, gate loop 2 and interface loop are shown and marked in red, black and blue dashed circles, respectively. METTL3 ZFD structural comparison among (**E**) *H. sapiens*, (**F**) *P. alecto* and (**G**) *R. aegyptiacus*. Black dashed circle highlights the areas of the hydrophobic residues shown in lines. The PDB IDs from which the MTD and ZFD of METTL3 were adapted are 5IL0 and 5ZOT, respectively.

Protein structural analysis of the human m6A main catalytic subunit METTL3 demonstrated that the methyltransferase domain (MTD) of the protein is located between amino acids 369 and 570; within this range, there were three functional loops, gate loop 1 (residues 396–410), gate loop 2 (residues 507–515) and an interface loop (residues 462–479). Residues in gate loops 1 and 2 are responsible for adenosine recognition of the S-adenosylmethionine (SAM) molecule, while the interface loop mediates the interaction between METTL3 and METTL14 [50]. The critical zinc finger domain (ZFD) of METTL3 identifies the m6A RRACH motif through hydrophobic residues, Ser315, Phe316, Cys320, Phe321 and His322. This domain consists of two zinc fingers, ZnF1 (residues 259–298) and ZnF2 (residues 299–336) [51]. Sequence alignment of the *P. alecto* and *R. aegyptiacus* METTL3, when compared to *H. sapiens*, showed 100% amino acid identity in the three loops of the MTD; furthermore, the two zinc fingers, including the critical RRACH motif identified residues, did not demonstrate any change when compared between humans and bats (Figure 5A). Likewise, all aforementioned structures were similar across the three mammalian species when the proteins were three-dimensionally structurally compared (Figure 5B–G).

The MTD of METTL14 (residues 117–402) contained an unusually long N-terminal extension (residues 116–165) mediating the METTL14–METTL3 interaction [50]. These residues were conserved among *H. sapiens*, *P. alecto* and *R. aegyptiacus* (Figure S7A), with no significant difference observed for the three species in the structural analysis (Figure S7B–D). The accessory m6A writer, WTAP, is a homodimer, each monomer consisting of four helices and three linkers. The N-terminus of the protein (residues 1–249) contains two helical chains, α3 (residues 148–176) and α4 (residues 177–249), respectively, that have residues Lys 155, 160, 192, 230 and Gly 170, which mediate the binding of the WTAP to the METTL3-METTL14 heterodimer [52]. The N-terminal domain of WTAP and key amino acids at the binding interface with METTL3-METTL14 were conserved in the alignment as well as the protein structural integrity (Figure S8A–D).

**Figure 6:**
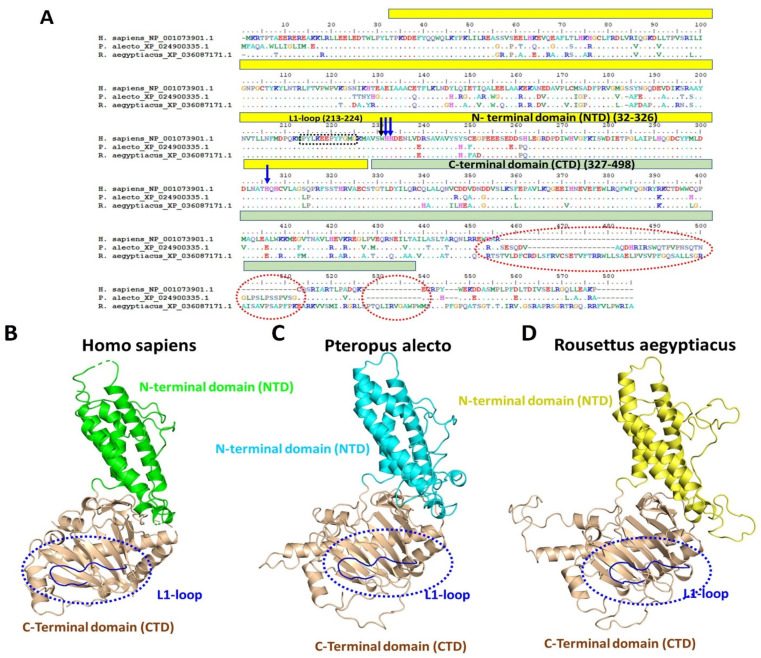
Alignment and protein structure comparisons of *H. sapiens*, *P. alecto* and *R. aegyptiacus* FTOs. (**A**) Multiple sequence alignment of the FTO of *H. sapiens*, *P. alecto* and *R. aegyptiacus*. NTD and CTD are marked with solid yellow and green rectangles, respectively. L1-loop residues are marked with a black rectangle. Areas of CTD deletion are marked in red dashed circles. The conserved Trp 230 and iron binding residues (His 231, Asp 233 and His 307) are demonstrated with black and blue arrows respectively. Overall structure comparison of FTO among (**B**) *H. sapiens*, (**C**) *P. alecto*, (**D**) *R. aegyptiacus.* Blue dashed circles are present around the conserved structure of the L1 loop of the proteins. The PDB ID from which FTO was adapted is 3LFM.

A pairwise comparison of the m6A FTO eraser between humans and the bats identified several amino acid substitutions across the two functional domains of the protein, the N-terminus (residues 32–326) and the C-terminal domain (CTD) that are responsible for carrying the protein catalytic “jelly roll” motif and iron binding coordinates, respectively [53]. Interestingly, some amino acids were conserved between *P. alecto* and *R. aegyptiacus* while others were uniquely specific to each bat species, although no variation could be seen among the three species at the 213–224 residues which formed the unique L1 loop responsible for the nucleic acid selectivity of the FTO [53]; similarly, the metal binding amino acids His 231, Asp 233 and His 307 and the Trp 230 that mediates the interaction between the L1 loop and the catalytic jelly roll motif were all conserved between the bats and humans (Figure 6A). It is worth noting that FTO had the highest amino acid variations of the m6A machinery analysed here; thus, the construction prediction of the two bat species’ FTOs when compared to the human structure showed visible distinctions among the three species; nevertheless, the configuration at the distinctive L1 loop remained undisrupted across the three mammals (Figure 6B–D).

An analysis of the eraser protein ALKBH5 structure showed that the Alk domain (residues 74–294) encompasses the conserved metal ion coordinate residues His-204, Asp-206 and His-266 of the protein forming the conserved H*X*(D/E) *XnH* motif. The ALKBH5 nucleotide recognition lid consists of two regions, Flip 1 (residues 117–129) and Flip 2 (residues 136–165). Furthermore, an additional region referred to as Flip 3, formed due to the disulfide bond between residues Cys-230 and Cys-267, gives ALKBH5 binding selectivity against unmethylated double-strand nucleic acids [54,55]. The alignment of the *H. sapiens*, *P. alecto* and *R. aegyptiacus* ALKBH5 was maintained; except for one, all residues formed the functional Alk domain (Figure S9A); nonetheless, the structure prediction showed variations at the metal binding residues and the characteristic Cys 230 and Cys 267 ones (Figure S9B–E).

**Figure 7:**
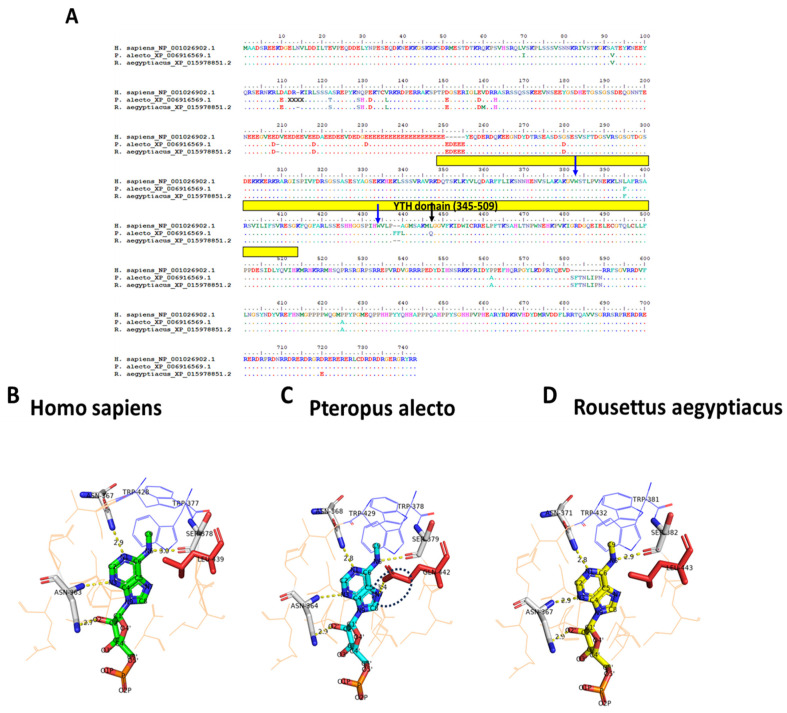
Amino acid alignment and m6A interaction of YTHDC1 of *H. sapiens*, *P. alecto* and *R. aegyptiacus*. (**A**) Alignment of *H. sapiens*, *P. alecto* and *R. aegyptiacus* YTHDC1 proteins. The YTH domain is highlighted with a solid yellow rectangle. The conserved aromatic residues Trp 377 and Trp 428 are highlighted with blue arrows. The substitution of Leu 439 is labelled with a black arrow. Three-dimensional structure comparison of YTHDC1 methylated RNA interacting area among (**B**) *H. sapiens*, (**C**) *P. alecto*, (**D**) *R. aegyptiacus*. A black dashed circle is used to highlight the extra hydrogen bond formed due to the Leu439Gln replacement in *P. alecto*. The PDB ID for YTHDC1 used here is 4R3I.

In a comparison of the *H. sapiens*, *P. alecto* and *R. aegyptiacus* m6A readers, the location of human YTH domains that identify the methylated RNA corresponded to residues 361–559, 380–579, 403–571, 345–509, 1277–1430 in YTHDF1/2/3 and YTHDC1/2, respectively [56,57,58,59]. An analysis of the human–Pteropodidae YTHDF1 alignment showed the lowest conservation compared to other readers (Figure S10). Additionally, compared to *P. alecto* and *H. sapiens*, inconsistent insertions and substitutions could be seen at the end of the YTHDF2 domain of *R. aegyptiacus* (Figure S11). One substantial feature of the YTH domain family is the presence of the aromatic cage which forms a hydrophobic pocket pinning the methylated adenine; this cage consists of Trp 411, Trp 465 and Trp 470 in YTHDF1, Tyr 418, Trp 432, Trp 486 and Trp 491 in YTHDF2, Trp492, Trp438 and Trp 497 in YTHDF3, Trp 377, Trp 428 and Leu 439 in YTHDC1, and Trp 1310, Trp 1360 and Leu 1365 in YTHDC2 [59,60,61,62]. Interestingly, all residues were conserved among humans and the two Pteropodidae (Figures S10–S13), except for the leucine 439 in the YTHDC1 of *P. alecto* which was significantly replaced by glutamine (Leu439Gln) (Figure 7A). Indeed, this replacement disrupted the m6A binding interaction in the black flying fox, compared with humans and the Egyptian fruit bat, forming an extra hydrogen bond between the NH2 of the glutamine side chain and the seventh nitrogen atom of the methylated adenine (Figure 7B–D).

**Figure 8:**
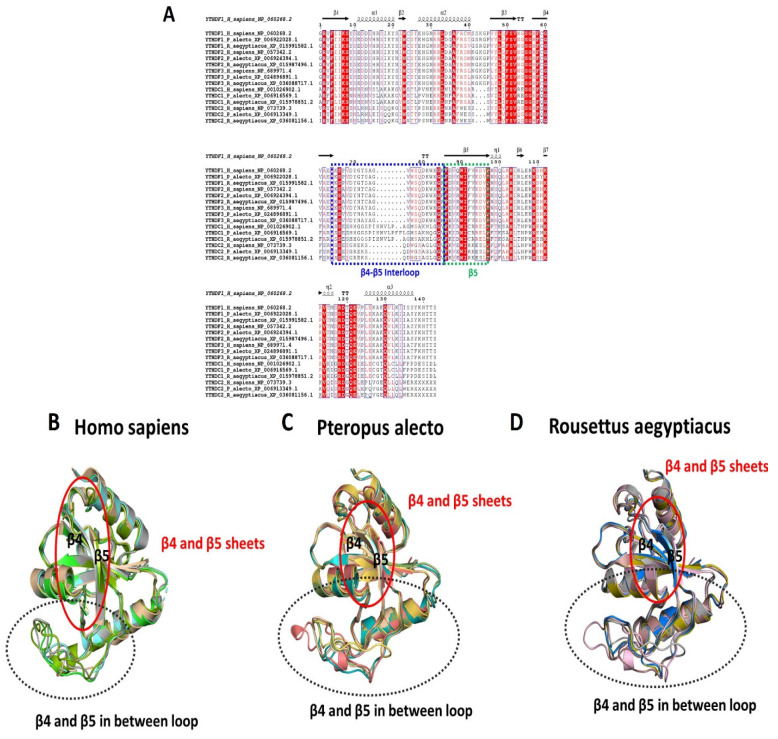
YTH domain family sequence alignment and superimposition comparisons among *H. sapiens*, *P. alecto* and *R. aegyptiacus*, (**A**) showing multiple amino acid sequence alignment of five reader proteins of *H. sapiens*, *P. alecto* and *R. aegyptiacus* along with the secondary structure. Sequences of β4-β5 interloops are highlighted in blue dashed boxes. Residues forming the β5 sheets are highlighted in green dashed boxes. Readers’ structural superimposition comparison among (**B**) *H. sapiens*, (**C**) *P. alecto*, (**D**) *R. aegyptiacus*. β4-β5 interloops are shown in black dashed circles. β4-β5 sheets are highlighted with solid red circles. PDB IDs of YTHDF1, YTHDF2, YTHDF3, YTHDC1 and YTHDC2 are 4RCI, 4WQN, 5ZOT, 4R3I and 6K6U, respectively.

Structurally, the YTH domain is nearly identical in all readers. The YTH domain YTHDF1, as an example, consists of the C-termini of β1, α1 and β2, the N-terminus of α2, and the loop between β4 and β5 [58]. However, in the multisequence alignment, the superimposition of the YTH domain of the five m6A readers, showed the same amino acid length among *H. sapiens*, *P. alecto* and *R. aegyptiacus* in the β5 sheet and an inconsistent length of the β4 and β5 between loops (Figure 8A); in the predicted structures, the length of the β5 sheets was noted to be shorter, while the β4 and β5 between loops were longer in the two bats than in humans (Figure 8B–D).

## 4. Discussion

The m6A epitranscriptomics mark has been known as a regulator of virus replication by acting as either a proviral or antiviral factor [63,64]. Owing to the importance of m6A during the virus replication and the fact that bats harbour zoonotic viruses perhaps more than any other mammalian order [65], here in this study, for the first time, we have provided a comprehensive structural and bioinformatic analysis of the m6A protein complex of *P. alecto* and *R. aegyptiacus*. Phylograms of all m6A proteins grouped *P. alecto* and *R. aegyptiacus* within the Pteropodidae clade in the mammalian cluster. However, FTO classified the order Chiroptera within the suborder Laurasiatheria as concluded previously [35,66,67], yet the presence of the bats as a monophyletic group within the order could point to unique sequence characteristics of the FTO in bats. Based upon the YTHDF3 tree, the Pteropodidae clade and Rhinolophidae (*R. ferrumequinum*) (Rhinolophidae) shared a common ancestor in the YTHDF3 reader tree, while a further common ancestor derived two families as well as the Hipposideridae (*H. armiger*); that was in opposition to [68] which elaborated that a common ancestor diverted Rhinolophidae and Hipposideridae while a further one diverted Rhinolophoidea and Pteropodidae.

The human genome size is about 3Gbp [69], while those of *P. alecto* and *R. aegyptiacus* are approximately 2 Gbp [35,36]. One putative goal of comparative genomics and genetic synteny is to understand gene function through the identification of the conserved genetic elements among species [70,71]. Indeed, genes correlated to m6A in humans were distributed on different autosomal chromosomes, and the fourth one was noted to carry two m6A-related genes; such a comparison cannot be executed in the black flying fox or the Egyptian fruit bat since their available genomes are mainly unplaced scaffolds. It is particularly interesting that many m6A-adjacent genes in the human chromosomal loci were the same in *P. alecto* and *R. aegyptiacus*. This could indicate the same implication of the bats’ m6A genes for the activity of their adjacent genes in a similar fashion to their human counterparts.

The bat1K genome initiative (https://bat1k.com/) aims to sequence the genomes of all bat species at the chromosomal level [72]. Until this project is accomplished, fully characterising the m6A machinery of *P. alecto* and *R. aegyptiacus* would provide genomic annotation and characterisation of these bats’ m6A genes compared to humans.

There is currently no protein structural or functional information available for the m6A readers, writers or erasers in the black flying fox or the Egyptian fruit bat. Therefore, to predict the structure and functions of bats’ m6A-assocated proteins, sequence alignment and homology modelling were performed using SWISS-MODEL as a reference comparative modelling method. This model was chosen due to its potential to consider the similarity and evolutionary relation between the target and the template protein [73,74]. Furthermore, the model offers the prediction of ligand interactions. The methylation reaction of m6A is catalysed in the nucleus by the heterodimer of METTL3-METTL14 [19]. Amino-acid-based alignment and the protein structure comparisons of METTL3 and METTL14 among *P. alecto*, *R. aegyptiacus* and *H. sapiens* could not reveal any dissimilarities in any of the functional regions, gate loops or domains. The N-terminus of the WTAP is responsible for the interaction with METTL3-METT14 through Lys residues at 155, 160, 192 and 230 and the Gly residue at 170 in the α3 and α4 helices [20,52]. We have observed that these amino acids were identical between the two bats and humans. Taken together, the results of the bats’ m6A writer complex could reveal the same catalytic reaction as in humans.

FTO and ALKBH5 are nonheme Fe(II)/2-oxoglutarate-dependent oxygenases that remove the methyl group from the adenosine [23]. In the human–bat alignments, the FTO had, among all other m6A proteins, the highest amino acid variations. Several insertions and substitutions particularly in the CTD of *P. alecto* and *R. aegyptiacus* carry the metal binding amino acids, yet the coordinates for metal binding have not changed in the two Chiroptera when compared to humans. Intriguingly, some amino acids in CTD were different between the two bats, as shown in the alignment and the protein structure comparison. These variations might affect the protein function in both species, although further confirmation is warranted. The vital L1 loop responsible for the selection of methylated single-strand nucleic acid remained unchanged. This still could point to the same nucleic acid selectivity pattern in the two bats and humans. Except for one amino acid alteration in the catalytic motif of ALKBH5, no noticeable mutations were observed. Despite this high conservation, the structure prediction of ALKBH5 in the black flying fox and the Egyptian fruit bat showed a misconfiguration at the iron recognition amino acids and the disulfide Cys-230-Cys-267 regions.

In humans, there are five YTH domain readers, and each contributes to a unique role in determining the metabolic fate of modified mRNAs [75]. An aromatic cage that holds the adenine moiety of the m6A-containing mRNA is a distinctive feature of this family [58]. Amino acid alignment of the human and the two bat readers showed different variability, where substitutions, insertions and deletions could be seen across the five proteins. Only one critical amino acid change was noted in the m6A adenine moiety binding pocket of the YTHDC1, which was the replacement of 439 leucine in *H. sapiens*, and its homologue in *R. aegyptiacus* with glutamine in *P. alecto*. This amino acid is responsible for the vital hydrophobic interaction with m6A within the cage [76]. However, the crystal structure of the human YTH domain of YTHDC1 showed three additional hydrogen bonds interacting with the methylated adenine nucleobase. These are constructed from the carbonyl oxygen main chain of Ser 378, the amino group of the Asn 367 side chain and the main chain NH2 group of Asn 363, which interact with sixth, first and third nitrogen atoms of the methylated nucleotide, respectively. Additionally, a fourth hydrogen bond could be seen between the C2′-ribosyl hydroxyl oxygen of m6A and the side chain of Asn 363 [59]. Interestingly, the alternation of the hydrophobic amino acid (leucine at 439 position) in *H. sapiens* and its analogue in *R. aegyptiacus* into the hydrophilic glutamine in *P. alecto* impacted the m6A interaction in the black flying fox. The predicted structure of *P. alecto* showed an extra hydrogen bond between the NH2 side chain group of the altered amino acid and the seventh nitrogen atom of the methylated adenine. Future studies in this area are warranted to define if this affects the m6A binding ability of the YTHDC1 protein of *P. alecto*.

The YTH domain is nearly conserved in all readers, and the binding pocket of the YTHDF1, as an example, is formed by the C-termini of β1, α1 and β2, the N-terminus of α2, and the loop between β4 and β5 [58]; however, the superimposition of YTH domains of the YTHF proteins in the two bats showed shorter β5 sheets and longer β4-β5 between loops compared to humans, which could not be seen when the proteins of the three species were aligned together.

## 5. Conclusions

Homology modelling and bioinformatics are powerful tools for gaining insights into m6A machinery; however, it is important to keep in mind that they are also error-prone. The study of the m6A machinery in bats is one of the key research problems that needs to be clarified. Our analysis of m6A proteins in *P. alecto* and *R. aegyptiacus* has highlighted many critical considerations, for example, whether the predicted conservations of the m6A writer complex or the aromatic amino acid variation in the YTHDC1 readers in the *P. alecto* would have an impact on the actual role of m6A machinery in bats. Such aspects need to be explored through further studies establishing the structures and functions of bats’ m6A proteins. Furthermore, upcoming state-of-the-art research studies are warranted to provide greater insights into the role of m6A RNA modification in regulating the bat–virus interactome.

## Figures and Tables

**Table 1:** Accession numbers for sequences of *H. sapiens*, *P. alecto* and *R. aegyptiacus* used in this study.

Protein	*H. sapiens*	*P. alecto*	*R. aegyptiacus*
METTL3	NP_062826.2	XP_006918390.1	XP_016021052.1
METTL14	NP_066012.1	XP_006917799.1	XP_016000318.1
WTAP	NP_001257460.1	XP_006915095.1	XP_016008204.1
ALKBH5	NP_060228.3	XP_006908504.1	XP_016000686.1
FTO	NP_001073901.1	XP_024900335.1	XP_036087171.1
YTHDF1	NP_060268.2	XP_006922028.1	XP_015991582.1
YTHDF2	NP_057342.2	XP_006924394.1	XP_015987496.1
YTHDF3	NP_689971.4	XP_024896891.1	XP_036088717.1
YTHDC1	NP_001026902.1	XP_015978851.2	XP_006916569.1
YTHDC2	NP_073739.3	XP_006913349.1	XP_036081156.1

**Table 2:** NCBI genome and Ref Seq assembly numbers used in this study.

Organism	NCBI Genome and Ref Seq Assembly
*H. sapiens*	GRCh38.p14, GCF_000001405.40
*P. alecto*	ASM32557v1, GCF_000325575.1
*R. aegyptiacus*	mRouAeg1.p, GCF_014176215.1

**Table 3:** Protein Data Bank (PDB) identification codes used in this study.

Protein Name	PDB Identification Code (ID)
METTL3 (MTD)	5IL0
METTL3 (ZFD)	5ZOT
METTL14 (MTD)	5k7u
WTAP (N-terminus)	7VF5
ALKBH5	4NJN
FTO	3LFM
YTHDF1	4RCI
YTHDF2	4WQN
YTHDF3	5ZOT
YTHDC1	4R3I
YTHDC2	6K6U

## Data Availability

The original contributions presented in the study are included in the article/Supplementary Materials, further inquiries can be directed to the corresponding author.

## Supplementary Materials

The following supporting information can be downloaded at https://www.mdpi.com/article/10.3390/genes15111361/s1: Figure S1: Phylogenetic trees of METTL3 and METTL14 orthologues. (A) The evolutionary relationship of the METTL3 orthologues. (B) The evolutionary relationship of the METTL14 orthologues. *P. alecto* and *R. aegyptiacus* are marked in solid diamond and cube shapes, respectively. Figure S2: Phylogenetic trees of WTAP and ALKBH5 orthologues. (A) The evolutionary relationship of the WTAP orthologues. (B) The evolutionary relationship of the ALKBH5 orthologues. *P. alecto* and *R. aegyptiacus* are marked in solid diamond and cube shapes, respectively. Figure S3: Phylogenetic trees of YTHDF1 and YTHDF2 orthologues. (A) The evolutionary relationship of the YTHDF1 orthologues. (B) The evolutionary relationship of the YTHDF2 orthologues. *P. alecto* and *R. aegyptiacus* are marked in solid diamond and cube shapes, respectively. Figure S4: Phylogenetic trees of YTHDC1 and YTHDC2 orthologues. (A) The evolutionary relationship of the YTHDC1 orthologues. (B) The evolutionary relationship of the YTHDC2 orthologues. *P. alecto* and *R. aegyptiacus* are marked in solid diamond and cube shapes, respectively. Figure S5: Identity matrices of METTL14, WTAP, ALKBH5, YTHDF3, YTHDC1 and YTHDC2 orthologues. (A) The identity matrix scores of the METTL14 orthologues. (B) The identity matrix scores of WTAP orthologues. (C) The identity matrix scores of ALKBH5 orthologues. (D) The identity matrix scores of YTHDF3 orthologues. (E) The identity matrix scores of YTHDC1 orthologues. (F) The identity matrix scores of YTHDC2 orthologues. *P. alecto* and *R. aegyptiacus* are marked in solid diamond and cube shapes, respectively. Figure S6: Genetic synteny comparisons of WTAP, YTHDF3, YTHDC1 and YTHDC2 of *H. sapiens*, *P. alecto* and *R. aegyptiacus*. (A) Genetic synteny of the WTAP among *H. sapiens*, *P. alecto* and *R. aegyptiacus*. (B) Genetic synteny of the YTHDF3 among *H. sapiens*, *P. alecto* and *R. aegyptiacus*. (C) Genetic synteny of the YTHDC1 among *H. sapiens*, *P. alecto* and *R. aegyptiacus*. (D) Genetic synteny of the YTHDC2 among *H. sapiens*, *P. alecto* and *R. aegyptiacus*. Figure S7: Alignment and protein structure comparisons of METT14 of *H. sapiens*, *P. alecto* and *R. aegyptiacus*. (A) METTL14 amino acid sequence alignment of *H. sapiens*, *P. alecto* and *R. aegyptiacus*. The METTL14 MTD is labelled with a solid yellow rectangle. The N-terminal extension is highlighted in a blue dashed rectangle. METTL14 MTD structural comparison among (B) *H. sapiens*, (C) *P. alecto*, (D) *R. aegyptiacus* indicating conservation. The N-terminal extension is highlighted in red dashed circles. The PDB ID from which the MTD was adapted is 5k7u. Figure S8: Alignment and protein structure comparisons of WTAP *H. sapiens*, *P. alecto* and *R. aegyptiacus*, (A) WTAP amino acid sequence alignment among *H. sapiens*, *P. alecto* and *R. aegyptiacus*. The sequence of the N terminus is illustrated with a solid yellow rectangle. Sequences of α3 and 4 that bind with the METTL3-METTL14 heterodimer are highlighted with red and blue dashed rectangles, respectively. The conserved heterodimer-bound residues (Lys 155, 160, 192, 230 and Gly 170) are highlighted with an arrow. Structural comparison of the WTAP N terminus among (B) *H. sapiens*, (C) *P. alecto*, (D) *R. aegyptiacus* showing conservation. α3 and 4 are highlighted with red and blue dashed circles, respectively. The METTL3-METTL14-bound amino acids (Lys 155, 160, 192, 230 and Gly 170) are shown in lines. The PDB ID from which the N terminus of the WTAP was adapted is 7VF5. Figure S9: Alignment and protein structure comparisons of *H. sapiens*, *P. alecto* and *R. aegyptiacus* ALKBH5. (A) ALKBH5 alignment among *H. sapiens*, *P. alecto* and *R. aegyptiacus*. The AlkB domain is highlighted with a solid yellow rectangle. Blue, red and black dashed frames are used to identify Flips 1, 2 and 3, respectively. The His 204, Asp 206 and His 266 iron binding coordinates are shown with magenta arrows. The positions of cysteines 230 and 267 that form the disulfide bond are indicated with black arrows. Overall structure comparison of ALKBH5 among (B) *H. sapiens*, (C) *P. alecto*, (D) *R. aegyptiacus*. Blue, red and black dashed circle highlights are present around Flip 1, 2 and 3, respectively. Zoom-in view of areas of Flip3 and metal binding residues among (E) *H. sapiens*, (F) *P. alecto*, (G) *R. aegyptiacus.* Structural changes of Flip 3 and the iron binding coordinates are shown in lines and highlighted within black and magenta dashed circles, respectively. The PDB ID from which ALKBH5 was adapted is 4NJN. Figure S10: Alignment comparisons of *H. sapiens*, *P. alecto* and *R. aegyptiacus* YTHDF1. The conserved aromatic cage motifs (Trp 411, Trp 465 and Trp 47) are labelled with blue arrows. Figure S11: Alignment comparisons of *H. sapiens*, *P. alecto* and *R. aegyptiacus* YTHDF2. The YTH domain is marked with a solid yellow rectangle. The area of YTH domain variations is highlighted with a blue dashed circle. The conserved hydrophobic amino acids (Trp 432, Trp 486 and Trp 491) are labelled with blue arrows. Figure S12: Alignment comparisons of *H. sapiens*, *P. alecto* and *R. aegyptiacus* YTHDF3. The YTH domain is highlighted with a solid yellow rectangle. The conserved aromatic cage motifs (Trp492, Trp438 and Trp497) are labelled with blue arrows. Figure S13: Alignment comparisons of *H. sapiens*, *P. alecto* and *R. aegyptiacus* YTHDC2. The YTH domain is marked with a solid yellow rectangle. The conserved hydrophobic amino acids (Trp 1310, Trp 1360 and Leu 1365) are labelled with blue arrows.
