# Supplementary material for: Structural Analysis of Virus Regulatory N6-Methyladenosine (m6A) Machinery of the Black Flying Fox (Pteropus alecto) and the Egyptian Fruit Bat (Rousettus aegyptiacus) Shows Evolutionary Conservation Amongst Mammals
Source: Genes (Basel). 2024 Oct 23;15(11):1361. doi: 10.3390/genes15111361 (PMC11594476; doi:10.3390/genes15111361)
Supplement: Supplementary file 1 [file genes-15-01361-s001.zip › genes-3187987-supplementary.pdf]

Supplementary data

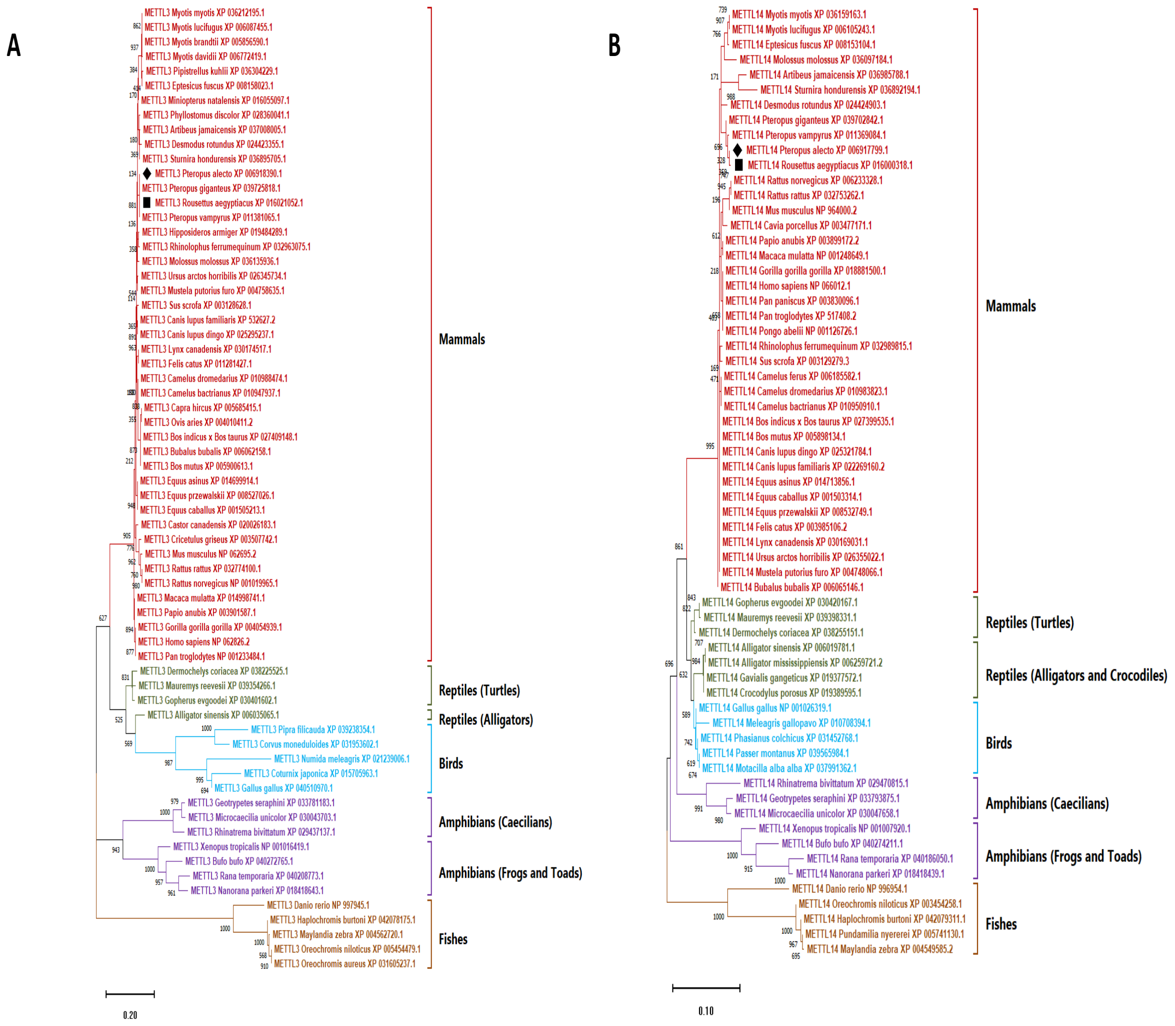

Figure S1. Phylogenetic trees of METTL3 and METTL14 orthologs. (A) showing the evolutionary relationship of the METTL3 orthologs. (B) showing the evolutionary relationship of the METTL14 orthologs. *P. alecto* and *R. aegyptiacus* are marked in solid diamond and cube shapes respectively.

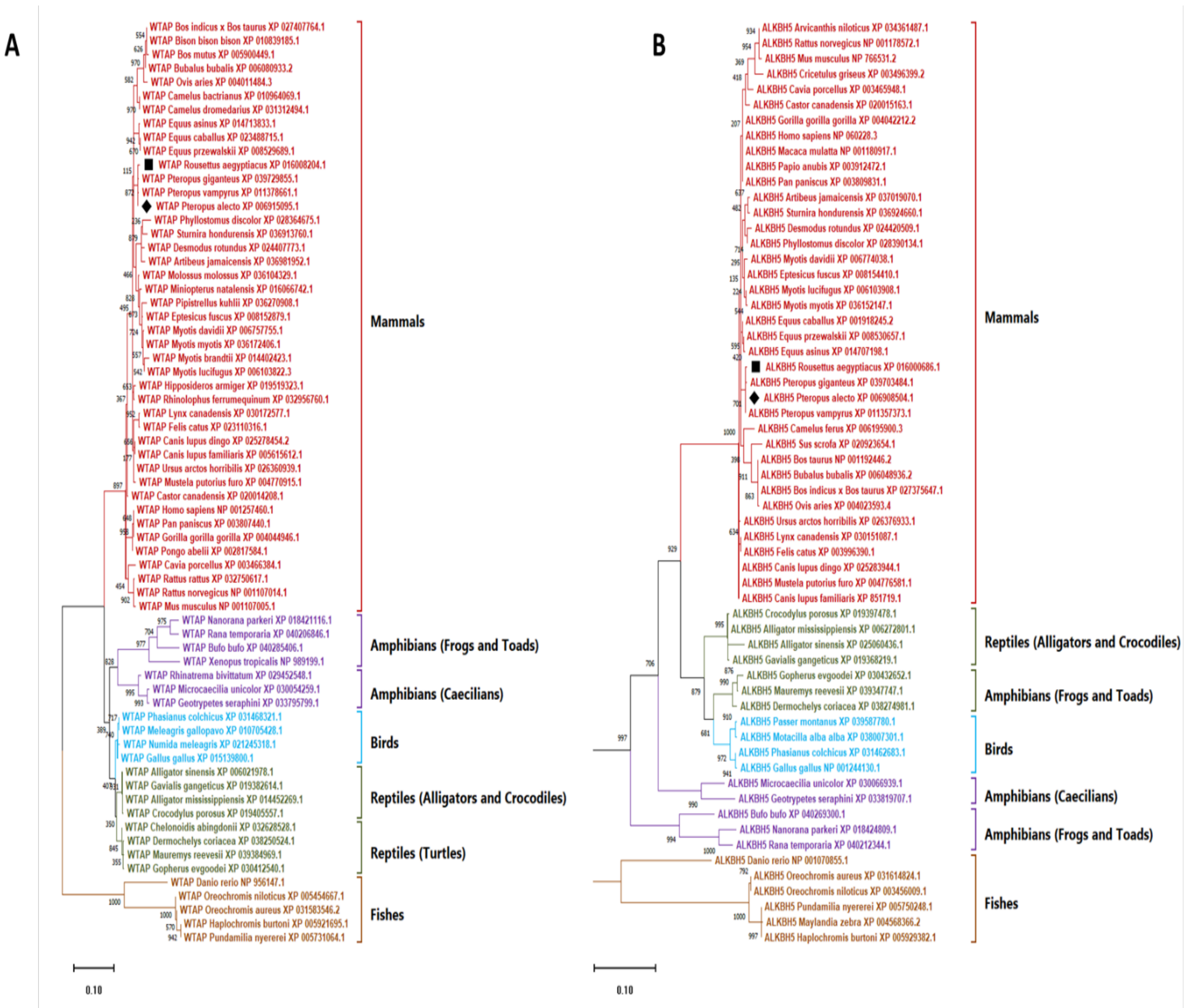

Figure S2. Phylogenetic trees of WTAP and ALKBH5 orthologs. (A) showing the evolutionary relationship of the WTAP orthologs. (B) showing the evolutionary relationship of the ALKBH5 orthologs. *P. alecto* and *R. aegyptiacus* are marked in solid diamond and cube shapes respectively.

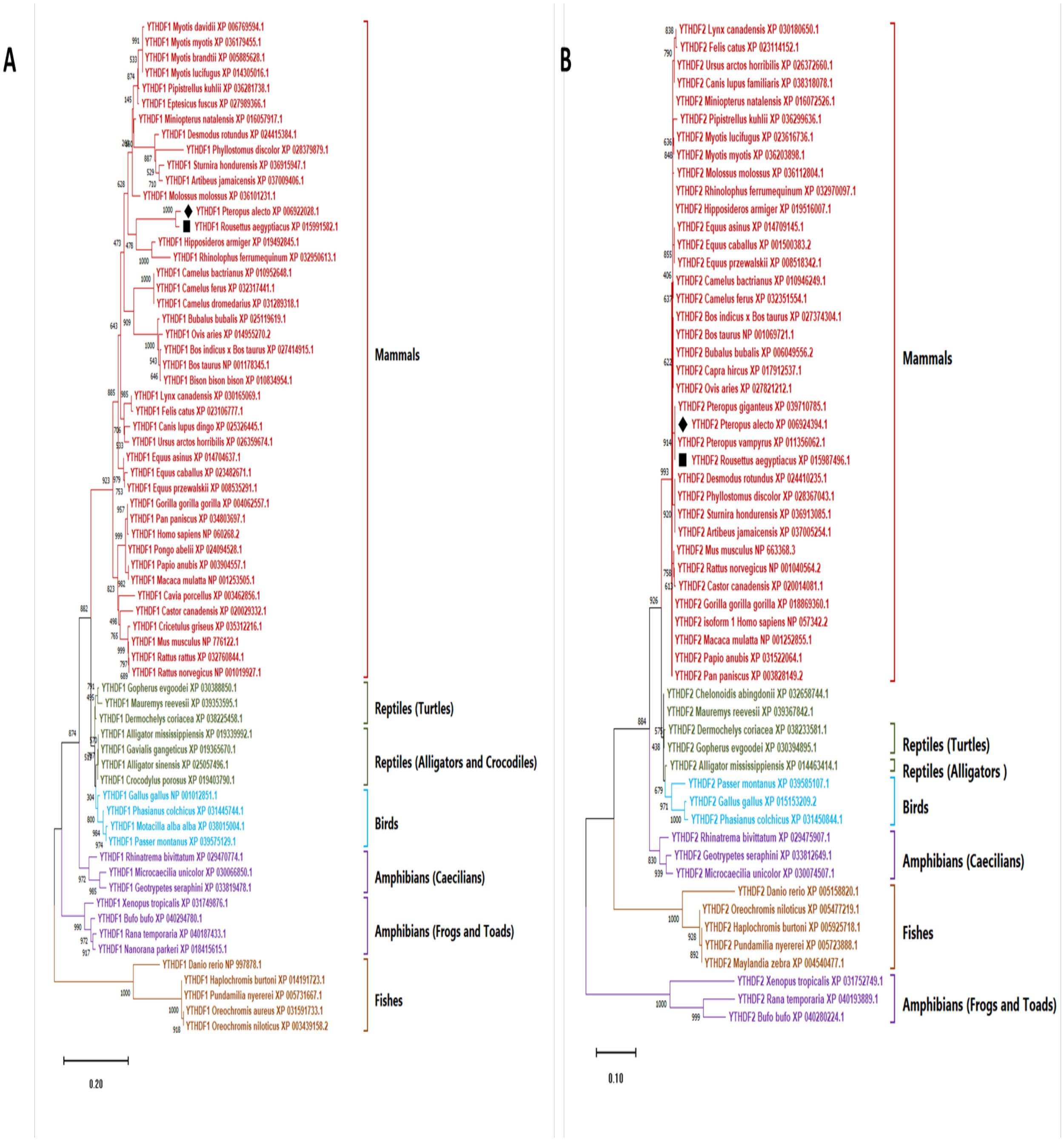

Figure S3. Phylogenetic trees of YTHDF1 and YTHDF2 orthologs. (A) showing the evolutionary relationship of the YTHDF1 orthologs. (B) showing the evolutionary relationship of the YTHDF2 orthologs. *P. alecto* and *R. aegyptiacus* are marked in solid diamond and cube shapes respectively.

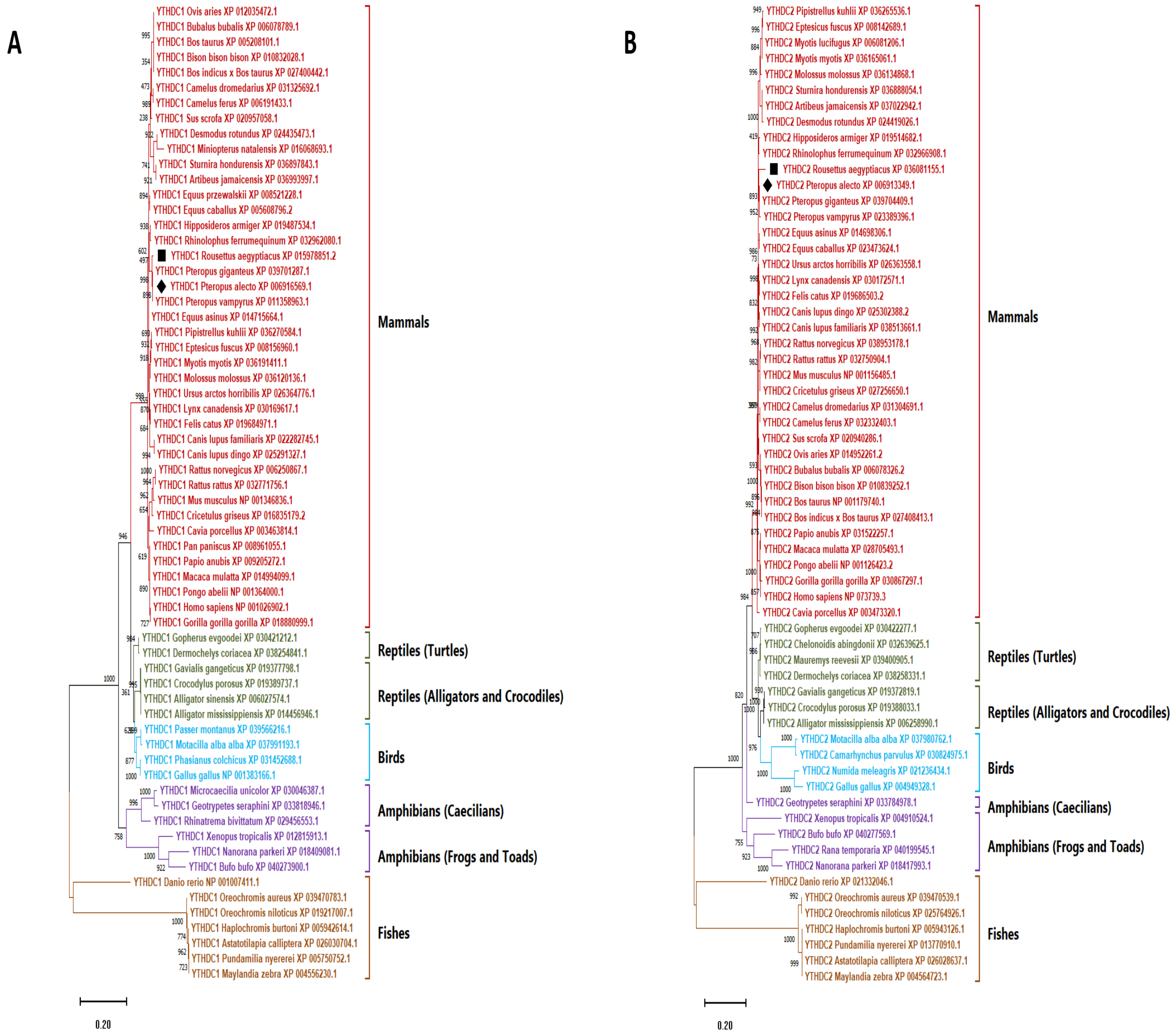

Figure S4. Phylogenetic trees of YTHDC1, and YTHDC2 orthologs. (A) showing the evolutionary relationship of the YTHDC1 orthologs. (B) showing the evolutionary relationship of the YTHDC2 orthologs. *P. alecto* and *R. aegyptiacus* are marked in solid diamond and cube shapes respectively.

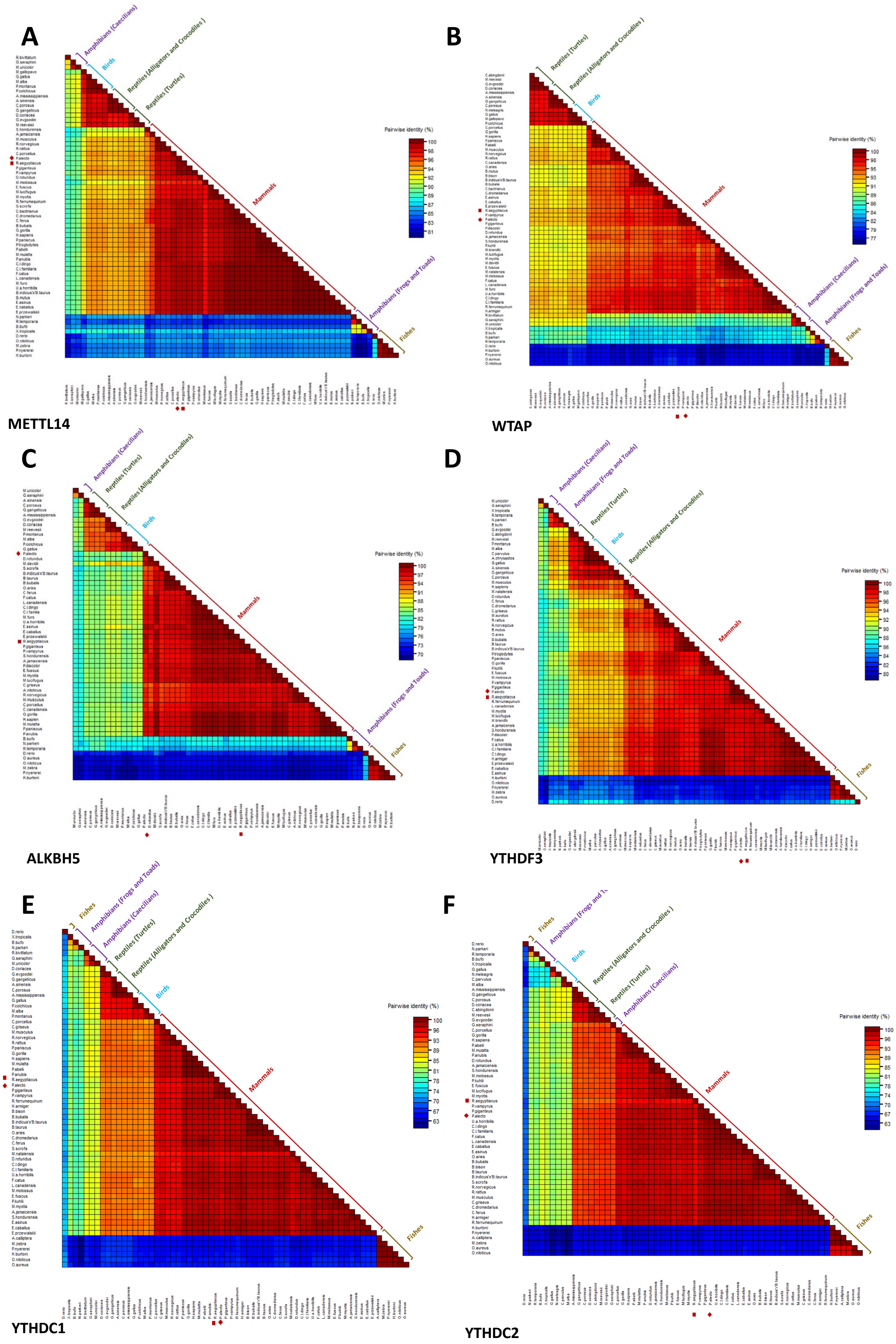
A
METTL14
B
WTAP
C
ALKBH5
D
YTHDF3
E
YTHDC1
F
YTHDC2
Pairwise identity (%)
Mammals
Birds
Fishes
Amphibians (Caecilians)
Amphibians (Frogs and Toads)
Reptiles (Turtles)
Reptiles (Alligators and Crocodiles)

Figure S5. Identity matrices of METTL14, WTAP, ALKBH5, YTHDF3, YTHDC1, and YTHDC2 orthologs. (A) showing the identity metrics scores of the METTL14 orthologs (B) showing the identity matrix scores of WTAP orthologs. (C) showing the identity matrix scores of ALKBH5 orthologs. (D), showing the identity matrix scores of YTHDF3 orthologs. (E) showing the identity matrix scores of YTHDC1 orthologs. (F) showing the identity matrix scores of YTHDC2 orthologs. *P. alecto* and *R. aegyptiacus* are marked in solid diamond and cube shapes respectively.

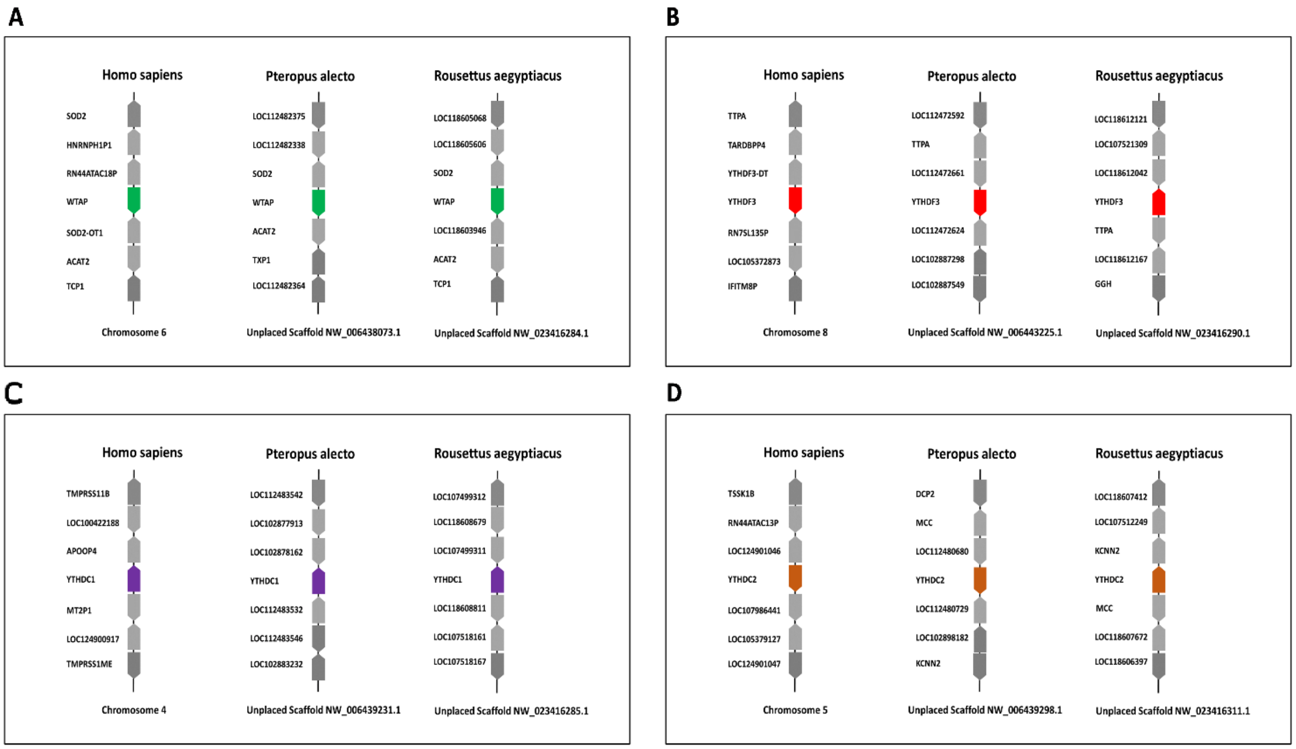

Figure S6. Genetic synteny comparisons of WTAP, YTHDF3, YTHDC1 and YTHDC2 of *H. sapiens, P. alecto*, and *R. aegyptiacus*. (A) Genetic synteny of the WTAP among *H. sapiens*, *P. alecto*, and *R. aegyptiacus*. (B) Genetic synteny of the YTHDF3 among *H. sapiens*, *P. alecto*, and *R. aegyptiacus*. (C) Genetic synteny of the YTHDC1 among *H. sapiens*, *P. alecto*, and *R. aegyptiacus*. (D) Genetic synteny of the YTHDC2 among *H. sapiens*, *P. alecto*, and *R. aegyptiacus*.

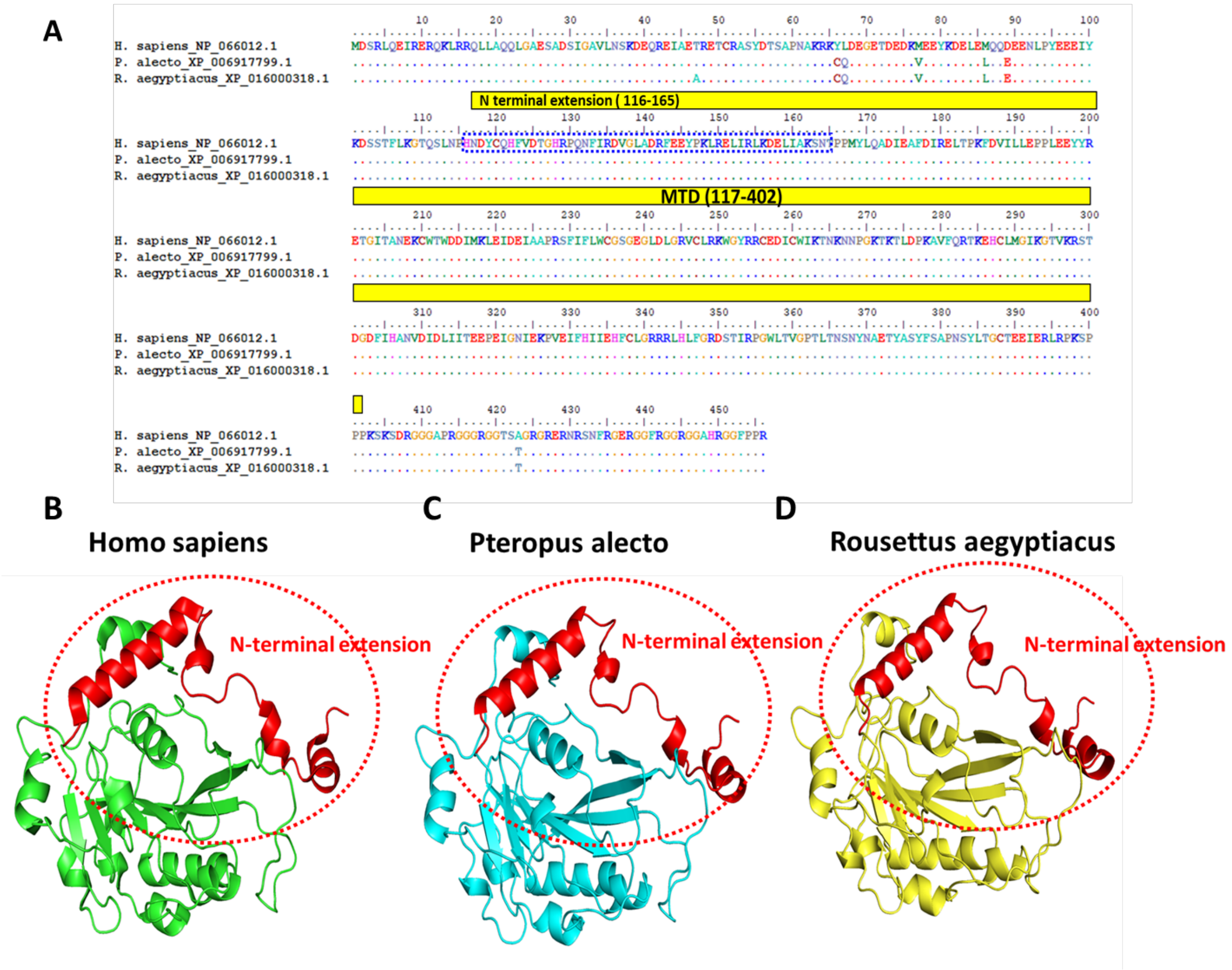

Figure S7. Alignment and protein structure comparisons of METT14 of *H. sapiens*, *P. alecto* and *R. aegyptiacus*. (A) METTL14 amino acid sequence alignment of *H. sapiens*, *P. alecto* and and *R. aegyptiacus*. The METTL14 MTD is labelled with a solid yellow rectangle. The N terminal extension is highlighted in a blue dash rectangle. METTL14 MTD structural comparison among (B) *H. sapiens* (C) *P. alecto* (D) *R. aegyptiacus* indicating conservation. The N terminal extension is highlighted in red dash circles The PDB ID from which the MTD was adapted is 5k7u.

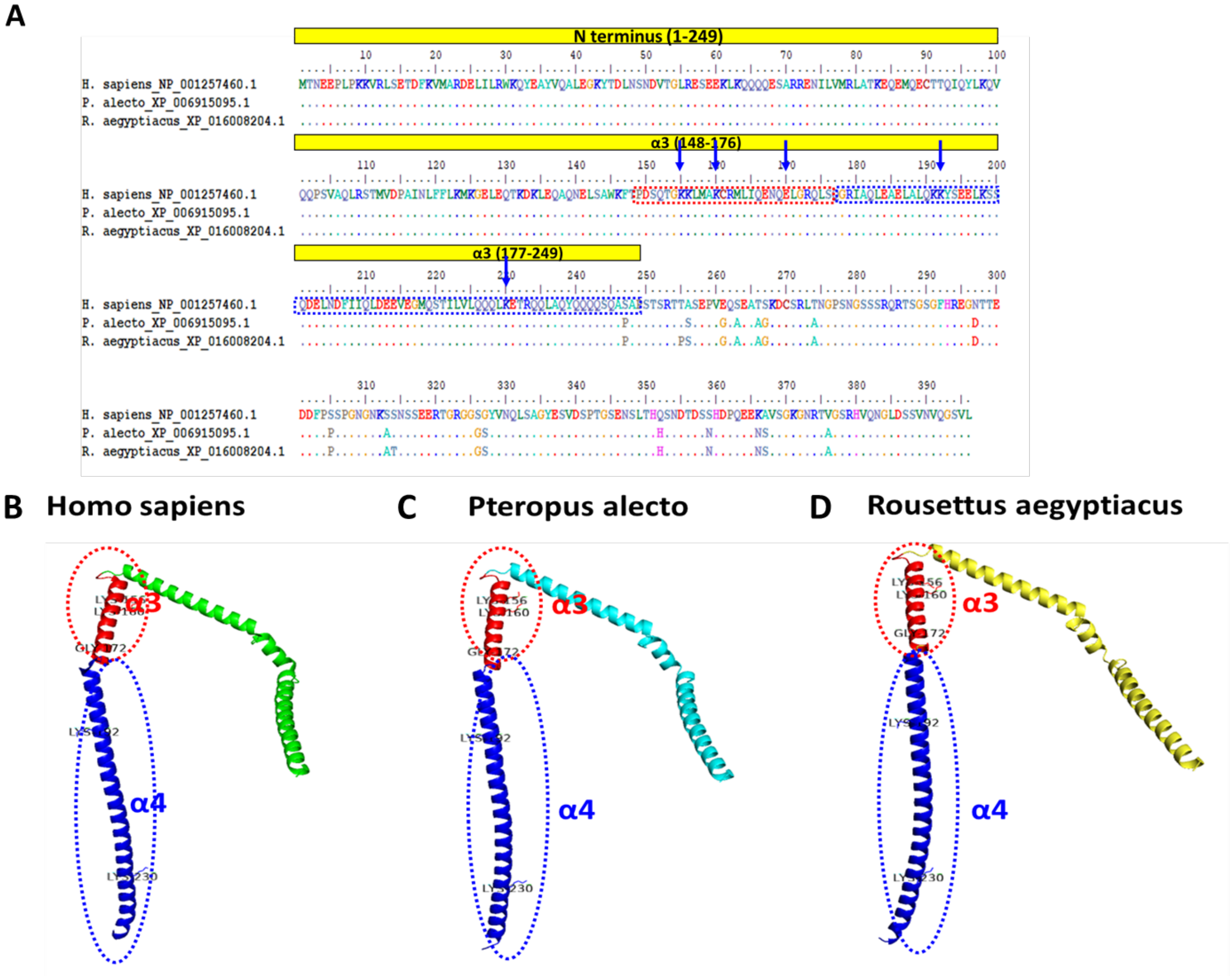

Figure S8. Alignment and protein structure comparisons of WTAP *H. sapiens, P. alecto* and *R. aegyptiacus*, (A) WTAP amino acid sequence alignment among *H. sapiens, P. alecto* and *R. aegyptiacus*. The sequence of the N terminus is illustrated with a solid yellow rectangle. Sequences of The α3 and 4 that bind with the METTL3-METTL14 heterodimer are highlighted with red and blue dash rectangles respectively. The conserved heterodimer bound residues (Lys 155, 160, 192, 230 and Gly 170) are highligthed with blow arrow. Structural comparison of the WTAP N terminus among (B) *H. sapiens* (C) *P. alecto* (D) *R. aegyptiacus* showing conservation. The α3 and 4 are highlighted with red and blue dash circles respectively. The METTL3-METTL14 bound aminoacids (Lys 155, 160, 192, 230 and Gly 170) are showed in line. The PDB ID from which the N terminus of the WTAP was adapted is 7VF5.

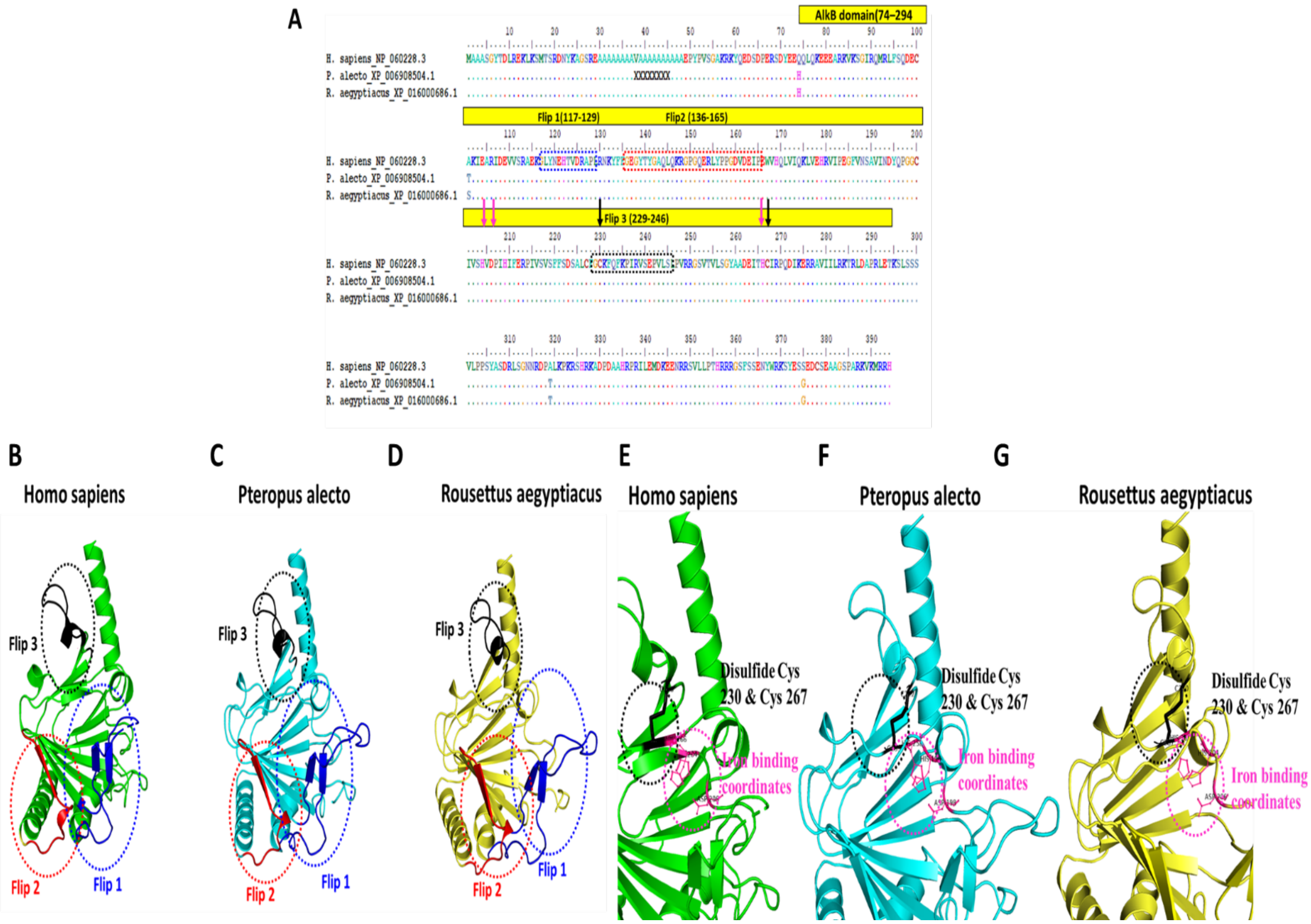

Figure S9. Alignment and protein structure comparisons of *H. sapiens, P. alecto* and *R. aegyptiacus* ALKBH5. (A) ALKBH5 alignment among *H.sapiens, P. alecto* and *R. aegyptiacus*. The AlkB domain is highlighted with a solid yellow rectangle. Blue, red, and black dashed frames are used to identify Flips 1, 2, and 3 respectively. The His 204, Asp 206, and His 266 iron binding coordinates are shown with magenta arrows. The positions of cysteines 230 and 267 that form the disulfide bond are indicated with black arrows. Overall structure comparison of ALKBH5 among (B) *H. sapiens* (C) *P. alecto* (D) *R. aegyptiacus*. Blue, red, and black dashed circle highlights are present around Flip 1, 2 and 3 respectively. Zoom in into areas of Flip3 and metal binding residues among (E) *H. sapiens* (F) *P. alecto* (G) *R. aegyptiacus.* Structural changes of Flip 3 and the iron binding coordinates are showed in lines and highlighted within black and magenta dashed circles respectively. The PDB ID from which ALKBH5 was adapted is 4NJN.

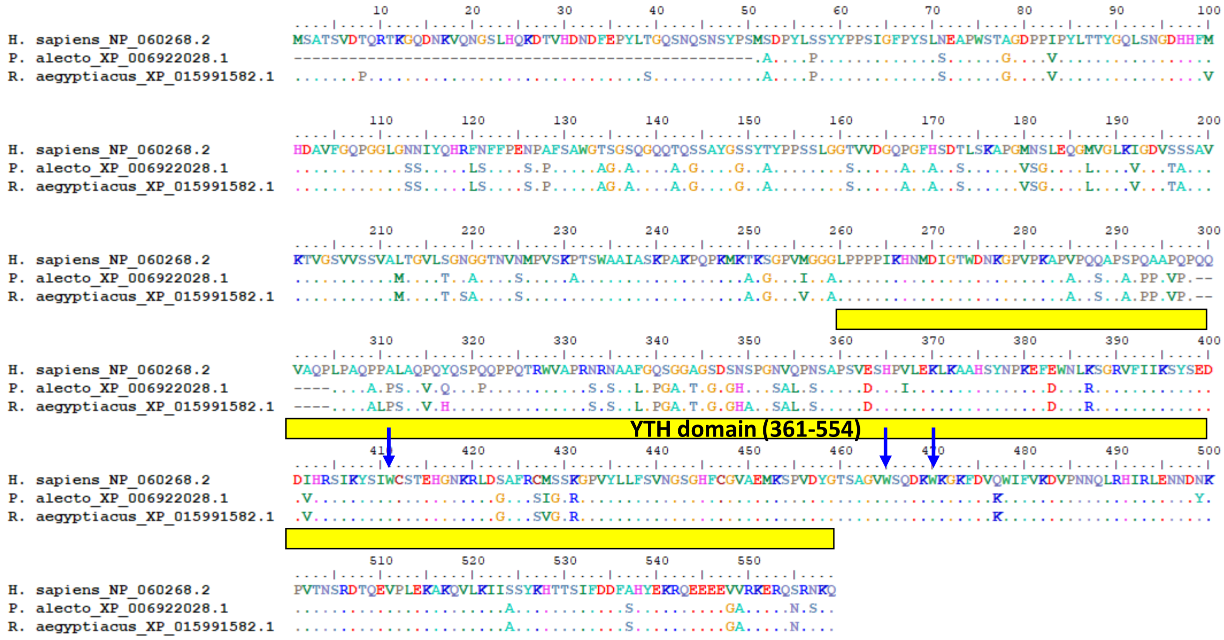

Figure S10. Alignment comparisons of *H. sapiens, P. alecto* and *R. aegyptiacus* YTHDF1. The conserved aromatic cage motifs (Trp 411, Trp 465, and Trp 47) are labelled with blue arrows.

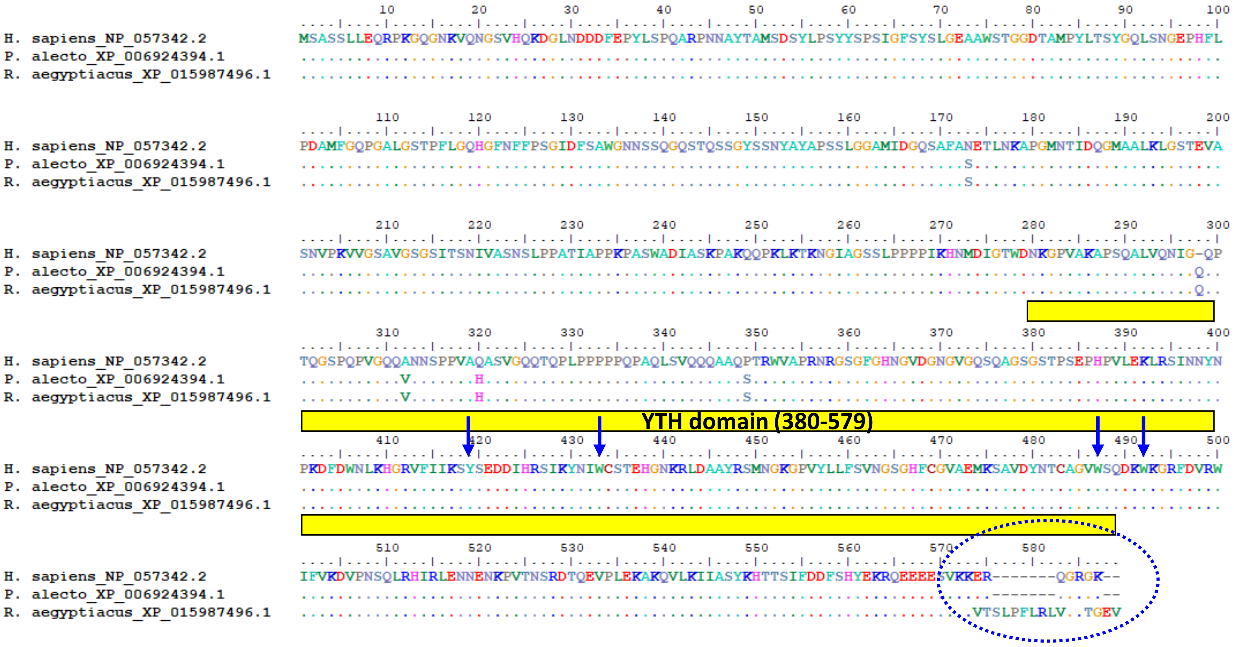

Figure S11. Alignment comparisons of *H. sapiens, P. alecto* and *R. aegyptiacus* YTHDF2. The YTH domain is marked with a solid yellow rectangle. The area of YTH domain variations is highlighted with a blue dashed circle. The conserved hydrophobic amino acids (Trp 432, Trp 486 and Trp 491) are labelled with blue arrows.

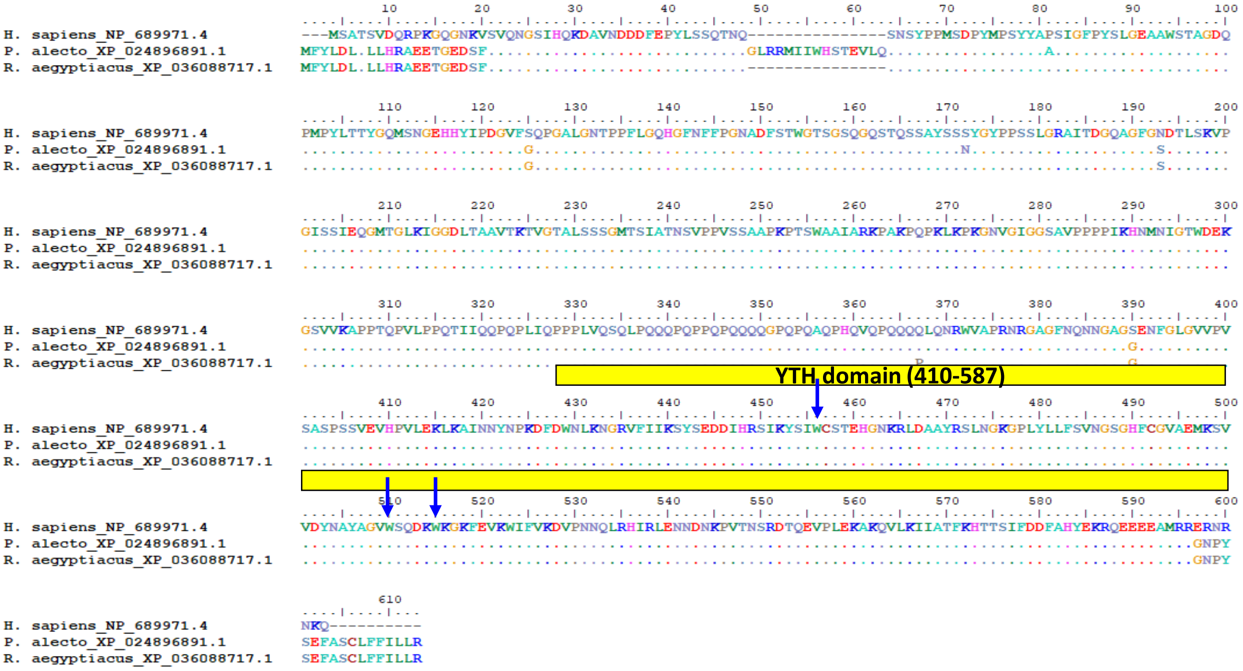

Figure S12. Alignment comparisons of *H. sapiens, P. alecto* and *R. aegyptiacus* YTHDF3. The YTH domain is highlighted with a solid yellow rectangle. The conserved aromatic cage motifs (Trp492, Trp438 and Trp497) are labelled with blue arrows.

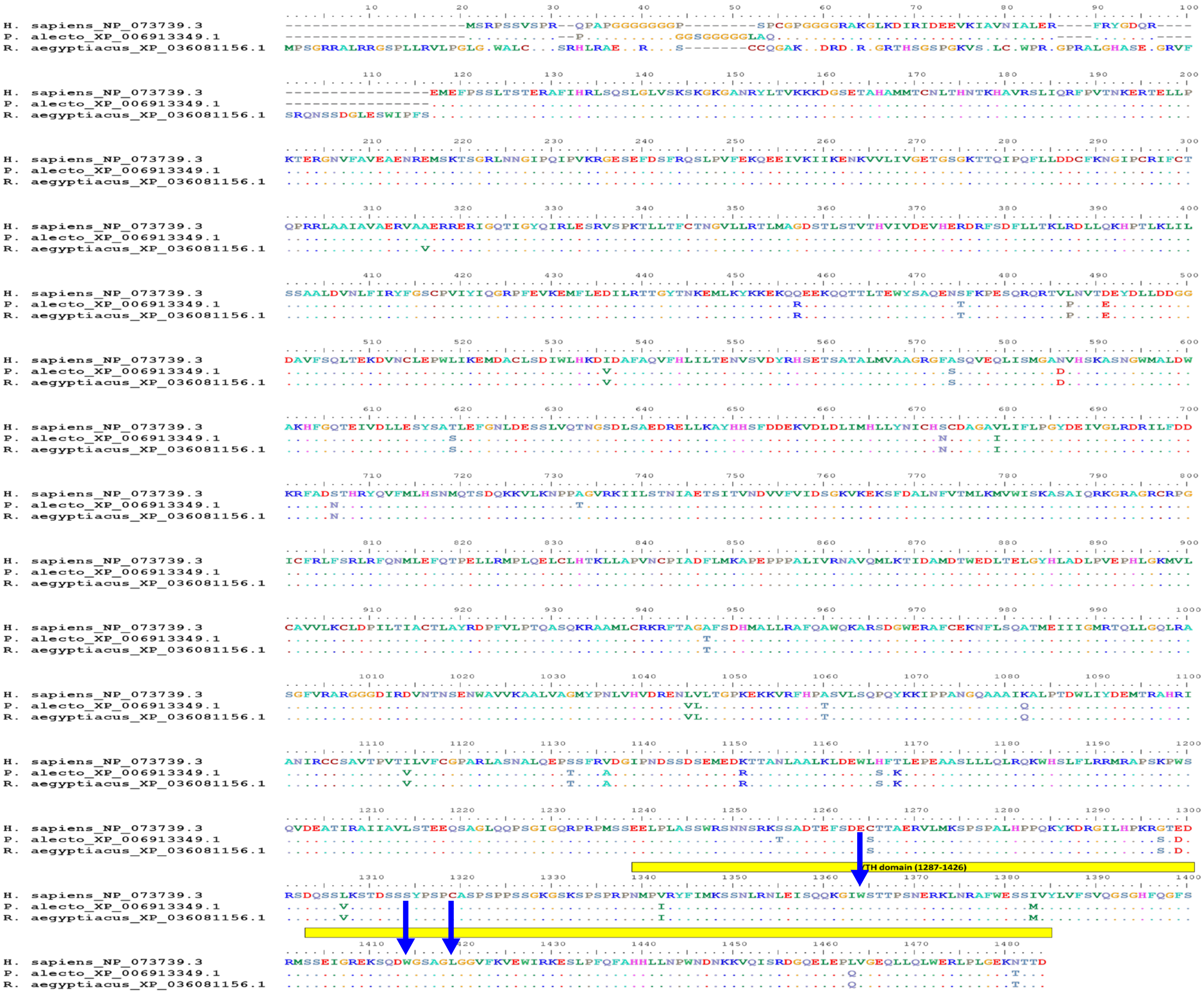

Figure S13. Alignment comparisons of *H. sapiens, P. alecto* and *R. aegyptiacus* YTHDC2. The YTH domain is marked with a solid yellow rectangle. The conserved hydrophobic amino acids (Trp 1310, Trp 1360 and Leu 1365) are labelled with blue arrows.
